# An Updated Organ-Based Multi-Level Model for Glucose Homeostasis: Organ Distributions, Timing, and Impact of Blood Flow

**DOI:** 10.3389/fphys.2021.619254

**Published:** 2021-06-01

**Authors:** Tilda Herrgårdh, Hao Li, Elin Nyman, Gunnar Cedersund

**Affiliations:** Department of Biomedical Engineering, Linköping University, Linköping, Sweden

**Keywords:** glucose homeostasis, glucose uptake, insulin signaling, mathematical modeling (medical), multi-level model

## Abstract

Glucose homeostasis is the tight control of glucose in the blood. This complex control is important, due to its malfunction in serious diseases like diabetes, and not yet sufficiently understood. Due to the involvement of numerous organs and sub-systems, each with their own intra-cellular control, we have developed a multi-level mathematical model, for glucose homeostasis, which integrates a variety of data. Over the last 10 years, this model has been used to insert new insights from the intra-cellular level into the larger whole-body perspective. However, the original cell-organ-body translation has during these years never been updated, despite several critical shortcomings, which also have not been resolved by other modeling efforts. For this reason, we here present an updated multi-level model. This model provides a more accurate sub-division of how much glucose is being taken up by the different organs. Unlike the original model, we now also account for the different dynamics seen in the different organs. The new model also incorporates the central impact of blood flow on insulin-stimulated glucose uptake. Each new improvement is clear upon visual inspection, and they are also supported by statistical tests. The final multi-level model describes >300 data points in >40 time-series and dose-response curves, resulting from a large variety of perturbations, describing both intra-cellular processes, organ fluxes, and whole-body meal responses. We hope that this model will serve as an improved basis for future data integration, useful for research and drug developments within diabetes.

## Introduction

A dysfunctional glucose homeostasis is a hallmark for both type 1 and type 2 diabetes mellitus (T1D and T2D). In T1D, the insulin-producing beta-cells are destroyed by the immune system. Since the other organs are unaffected, the treatment of T1D simply consists of insulin, taken via injections or insulin pumps. In T2D, the patient has both a reduced capacity to produce insulin and has developed a resistance to the hormone. This resistance appears in all of the three most metabolically active organs, which all respond to insulin: adipose tissue, muscle, and liver. Inside each of these organs, the response to insulin is governed by an interaction between intracellular signaling and metabolic networks. The resistance is spread between the organs, in ways which are not yet fully understood, but which involves numerous hormones, cytokines, and metabolites. To better understand this complex interaction, both in health and in disease, dynamic mathematical models are needed. Models for the top-level glucose homeostasis, involving a simple interaction between glucose and insulin, have been around for decades (Bergman et al., 1981). A first more advanced model (Dalla Man et al., 2007) was based on calculated flows of glucose and insulin between organs in response to a meal. A version of this model, trained on data from patients with type 1 diabetes, is approved by the Food and Drug Administration, FDA, for replacement of animal experiments in the approval of the algorithm inside new insulin pumps (Kovatchev et al., 2009). For more general applications involving T2D, the intracellular insulin resistance must be combined with the whole-body interactions. Such models are called multi-level models.

There have been several efforts to create multi-level models of glucose homeostasis, reviewed in e.g., (Nyman et al., 2012, 2016; Ajmera et al., 2013). One of the more comprehensive efforts is a series of non-linear mixed effects models (Jauslin et al., 2007; Silber et al., 2007, 2010) developed to describe plasma levels of glucose and insulin after different interventions for single patients with T2D. Another effort has developed a glucose homeostasis model, based partly on Dalla Man et al. (2007), to create a simulator to use in education and to simulate scenarios of disease (Maas et al., 2015). A third effort is the multi-level model of human glucose homeostasis we created 10 years ago (Nyman et al., 2011). This model contains the dynamic glucose-insulin interaction between organs in response to a meal, based on Dalla Man et al. (2007). In this model, we sub-divided the original insulin-responding uptake in a muscle and a fat component, and linked the fat tissue glucose uptake to intracellular insulin signaling data, coming from our own studies. This link was possible since insulin-stimulated glucose uptake can be measured both in isolated adipocytes and in organs. The adipocyte uptake is measured *in vitro* together with insulin signaling data; the organ-level uptakes are measured using isotopic labeling and/or arteriovenous (AV) difference data, which measures the difference between arterial and venous blood. Since the uptake measurements from isolated adipocytes should correlate with the AV difference-based uptake-measurements for fat tissue, one can build a translation from *in vitro* to *in situ*, in humans. However, neither this model, nor any of the previously mentioned multi-level models, have subdivided the glucose uptake into the individual contributions of all of the main insulin-responding and glucose-utilizing organs: adipose tissue, muscle, and liver.

**Figure 1:**
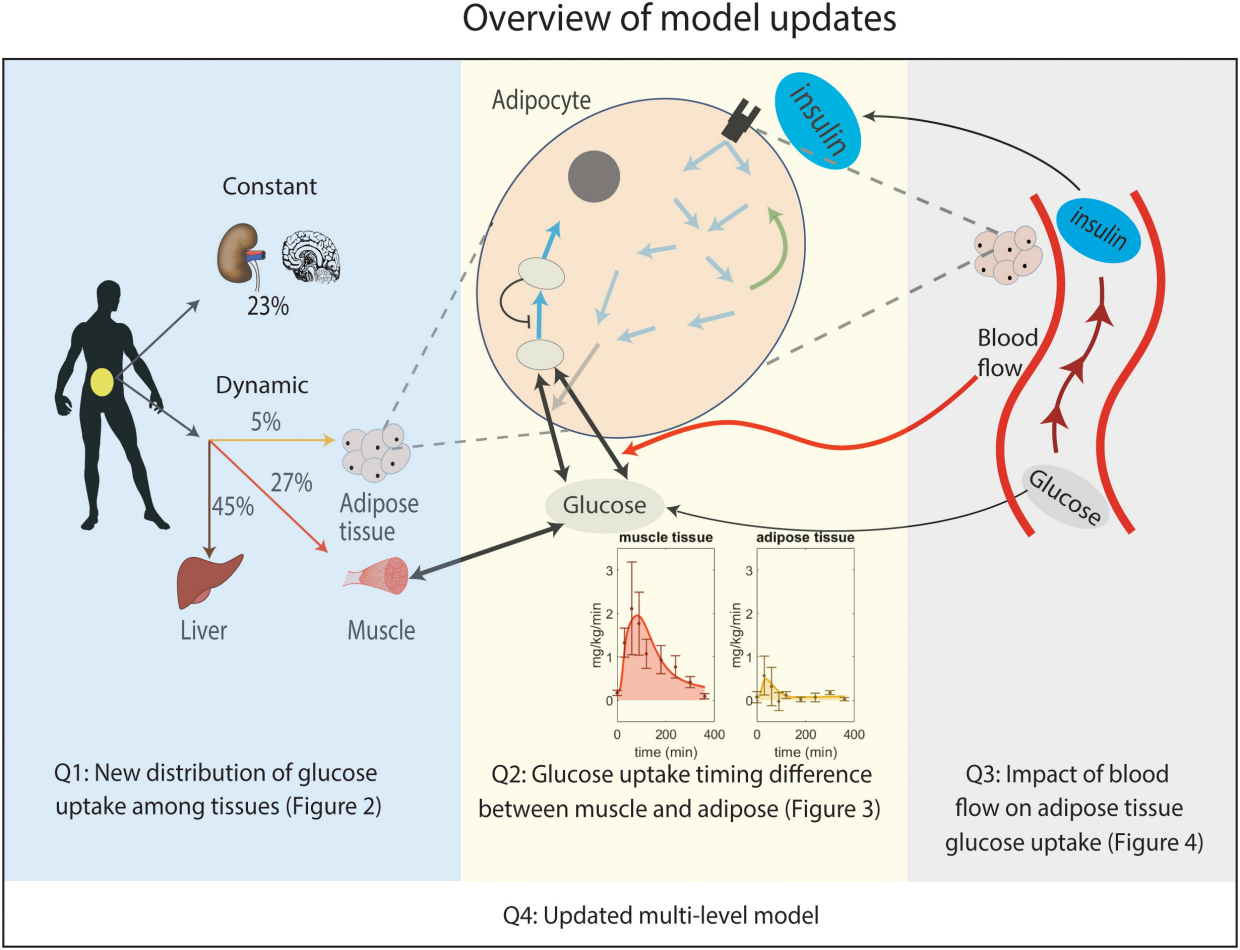
Overview of the improvements made to the original multi-level model. Q1: New distribution of postprandial glucose uptake among tissues in the whole-body level model. Q2: Implemented timing difference in glucose uptake between muscle and adipose tissue by adding phosphorylation of intracellular glucose in the adipocyte module for glucose uptake (non-shaded arrows in adipocyte, see Supplementary Figure 1 for details). Note that the addition of a detailed myocyte module is not needed to explain the considered data. Q3: Inclusion of the impact of blood flow on glucose uptake in adipose tissue. Q4: Merging Q1, Q2, and Q3 boxes together gives an updated multi-level metabolic model.

In this paper, we have updated the original multi-level connections in Nyman et al. (2011), and resolved three critical questions or issues (Q1-Q3), regarding the role of each of the metabolically active organs in glucose uptake (Figure 1). More specifically, we have explicitly included the liver in the model as a glucose-utilizing organ, in contrast to the original models, which only considered it as a glucose producing organ (Q1). Secondly, we have included a timing difference between muscle and adipose tissue glucose uptake in the response to a meal (Q2). Thirdly, we have updated the model to include the impact of blood flow on glucose uptake in adipose tissue (Q3). Finally, we merge these three improvements together with all of the other already published improvements described above, to an updated multi-level model (Q4). This model constitutes an updated view on the multi-level roles that each organ plays in glucose homeostasis, and allows for integration of future data for specific sub-systems into an integrated and more complete picture.

## Materials and Methods

### Glucose Dynamics in Plasma and Interstitial Tissue

The models are built up by ordinary differential equations (ODEs) in the standard form. All of the equations are given in the Supplementary Files, both as equations and as simulation files, and here we only describe the most central equations, relating to the changes done in this paper. The equations describing the dynamics of glucose in interstitial tissue (*G*_*t*_) and plasma (*G*_*p*_) are given by

(1) $$\begin{array}{l} \frac{d}{dt}\left(G_{t}\right)=-U_{id}+k_{1}\cdot G_{p}-k_{2}\cdot G_{t}\\ G_{t}\left(0\right)=130\left[mg/dl\right] \end{array}$$

(2) $$\begin{array}{l} \frac{d}{dt}\left(G_{p}\right)=EGP+Ra-E-U_{ii}-k_{1}\cdot G_{p}+k_{2}\cdot G_{t}\\ G_{p}\left(0\right)=178\left[mg/dl\right] \end{array}$$

where *U*_*id*_ is insulin and glucose dependent glucose uptake, i.e., in fat, muscle, and liver; where *U*_*ii*_ is insulin independent and constant glucose utilization, i.e., glucose uptake by organs such as brain and kidneys; where EGP is endogenous glucose production from the liver; where *Ra* is glucose rate of appearance from the intestine; where *E* is glucose excretion through the kidneys; and where *k*_1_ · *G*_*p*_ and *k*_2_ · *G*_*t*_ denotes the flux from plasma to intestines and back, respectively. Note that *G*_*t*_ and *G*_*p*_ are states, while *U*_*id*_, *U*_*ii*_, *k*_1_ · *G*_*p*_, *k*_2_ · *G*_*t*_, EGP, *Ra*, and *E* are the reaction rates that describe flows of glucose. Similarly, *k*_1_ and *k*_2_ are parameters—rate constants—which are constant over time.

### Insulin-Dependent and Dynamic Glucose Uptake

The above equations are identical to those in the original Dalla Man model (Dalla Man et al., 2007), and the change that was implemented in Nyman et al. (2011) was that *U*_*id*_ was sub-divided into a muscle and an adipose tissue part. We now sub-divide the insulin-dependent dynamic glucose uptake into three parts, i.e.,

(3) $$\begin{array}{l} U_{id}=U_{idm}+U_{idl}+U_{idf} \end{array}$$

where *U*_*idm*_, *U*_*idl*_, and *U*_*idf*_ denotes the uptake rates into the muscle, liver, and fat, respectively. All of these uptake descriptions have changed to same extent, so let us now go through them one by one.

### Glucose Uptake in Muscle

Glucose uptake in muscle is given by

(4) $$\begin{array}{l} U_{idm}=V_{mmax}\cdot \frac{G_{t}}{\left(K_{m}+G_{t}\right)} \end{array}$$

where *V*_*mmax*_ is the non-scaled maximal glucose uptake, and where *K*_*m*_ is the corresponding Michaelis-Menten parameter. The insulin-dependency of the glucose uptake is located in the expression for *V*_*mmax*_

(5) $$\begin{array}{l} V_{mmax}=part_{m}\cdot \left(V+V_{X}\cdot INS\right) \end{array}$$

where *part*_*m*_ is a scaling parameter to balance the uptake of the muscle with the other organs, where *V* is the basal rate of glucose utilization, and *V*_*X*_ is the maximum rate of glucose entering the tissue (here muscle) from the surrounding tissue, and where *INS* denotes the interstitial insulin concentration. So far, these equations for the muscle uptake are the same as in Nyman et al. (2011). In contrast, although *INS* is almost calculated in the same way as in Nyman et al. (2011), the parameters describing the rate of entry and the rate of degradation are now allowed to be different, i.e.,

(6) $$\begin{array}{l} \frac{d}{dt}\left(INS\right)=V_{1}-V_{2} \end{array}$$

(7) $$\begin{array}{l} V_{1}=k_{1}\cdot \left(I-I_{b}\right) \end{array}$$

(8) $$\begin{array}{l} V_{2}=k_{2}\cdot INS \end{array}$$

where *V*_1_ and *V*_2_ describe the rate of transport from the plasma and the rate of degradation, with corresponding rate constants *k*_1_ and *k*_2_, respectively; where *I*_*b*_ denotes the basal plasma insulin concentration; and where *I* denotes the insulin concentration in plasma.

### Glucose Uptake in the Liver

The liver was not included in the previous models, and thus its equations are new. They are similar to the equations for muscle, i.e.,

(9) $$\begin{array}{l} U_{idl}=V_{lmax}\cdot \frac{G_{t}}{\left(K_{l}+G_{t}\right)} \end{array}$$

where *K*_*l*_ is a Michaelis-Menten constant, and where *V*_*lmax*_ represents the maximum rate of glucose utilization in the liver. Just as for the equations for muscle, the insulin dependence is incorporated into the expression for *V*_*lmax*_, which is given by

(10) $$\begin{array}{l} V_{lmax}=part_{l}\cdot \left(V+V_{X}\cdot INS\right) \end{array}$$

where *part*_*l*_ represent the relative glucose utilization of the liver in comparison with other tissues.

Note that the insulin-dependency of the liver glucose uptake is described as being direct, while in reality this dependency is indirect. Glucose uptake in the liver is done via the GLUT2 transporter, which is not regulated by insulin. In contrast, the glucose uptake in muscle and adipose tissue is done by the GLUT4 transporter, which is regulated by insulin. In the liver, insulin instead indirectly effects glucose uptake by up-regulating intracellular glucose phosphorylation and utilization. However, since the model is lacking intracellular reactions, this indirect effect present in the liver is approximated in the same way as the direct effect for the muscle. Note, finally, that the endogenous glucose production (EGP) in the liver is also regulated by insulin, and that this is described as a separate process, in the same way as in Nyman et al. (2011).

### Glucose Uptake and Metabolism in Adipose Tissue

Glucose uptake in the adipose tissue is the most advanced part of the model, since it is determined by intracellular processes, both regarding metabolism and regarding insulin signaling. The ultimate calculation of the uptake is given by the following expression

(11) $$\begin{array}{l} U_{idf}=part_{f}\cdot \left(V_{in}-V_{out}\right) \end{array}$$

where *part*_*f*_ is a scaling parameter, and where *V*_*in*_ and *V*_*out*_ describe the rate of glucose transport into, and out of, the cell, respectively. These two fluxes are given by

(12) $$\begin{array}{l} V_{in}=p_{4}\cdot G_{t}\cdot {INS}_{f,e} \end{array}$$

(13) $$\begin{array}{l} V_{out}=p_{3}\cdot Glu_{in}\cdot {INS}_{f,e} \end{array}$$

where *p*_3_ and *p*_4_ are transport parameters, where *Glu*_*in*_ is the amount of intracellular glucose, and where *INS*_*f,e*_ is the effect of insulin on these transport rates. These two equations show that the glucose uptake in the fat tissue depends on both intracellular metabolism, which alters the value of *Glu*_*in*_, and the intracellular signaling, which alters the value of *INS*_*f,e*_. The intracellular metabolism incorporates the first two steps of glycolysis, i.e., the steps involving intracellular glucose-6-phosphate (*G*_6*P*_). The equations are given by

(14) $$\begin{array}{l} \frac{d}{dt}\left({Glu}_{in}\right)=p_{1}\cdot \left(V_{in}-V_{out}\right)-V_{G6P} \end{array}$$

(15) $$\begin{array}{l} \frac{d}{dt}\left(G6P\right)=V_{G6P}-V_{met} \end{array}$$

(16) $$\begin{array}{l} V_{met}=p2\cdot G6P \end{array}$$

(17) $$\begin{array}{l} V_{G6P}=V_{G6Pmax}\cdot \frac{{Glu}_{in}}{\left(k_{gluin}+{Glu}_{in}\right)}\cdot \frac{1}{\left(k_{G6P}+G6P\right)} \end{array}$$

where *p*1 and *p*2 are rate constants, *V*_*G*6*Pmax*_ is the maximum rate of phosphorylation of glucose, and where *V*_*gluin*_ and *k*_*G*6*P*_ are Michaelis-Menten parameters for glucose phosphorylation. The intracellular insulin signaling is the same as in Brännmark et al. (2013), and it starts with insulin binding to the receptor (Supplementary Equation 26), and ends with translocation of the GLUT4 transporter to the membrane (Supplementary Equations 46 and 45). What is new compared to Nyman et al. (2011)and Brännmark et al. (2013) instead concerns the usage of the GLUT4 transporter to calculate the resulting impact on glucose uptake, *INS*_*f,e*_. In our updated model, this insulin effect is given by

(18) $$\begin{array}{l} INS_{f,e}=nC\cdot \left(k8\cdot \frac{GLUT4m}{pf}+\frac{GLUT1}{pf}+{bfe}_{f}\right) \end{array}$$

where *GLUT*4*m* is the amount of GLUT4 in the membrane, where *GLUT*1 is the amount of GLUT1 in the membrane, and where *bfe*_*f*_ is the effect of blood flow; *nC*, *k*8, and *pf* are parameters. The GLUT4 and GLUT1 terms corresponds to the transport via the two glucose transporters, and *bfe*_*f*_ was introduced in Nyman et al. (2011) as a scaling parameter between the data from the *in vitro* setting studying isolated adipocytes, and the *in situ* setting, where the adipose tissue is still located in the human body. In other words, the blood flow effect is not there when simulating *in vitro* experiments. In Nyman et al. (2011), this difference in insulin effect was hypothesized to be dependent on blood flow, and in this paper, we show that such an impact on blood flow is indeed present. If one does not have data for the blood flow, the model will set *bfe*_*f*_ to a constant value, and if there is data for blood flow, we propose to use the new model described in the next section.

### Equations for the Impact of Blood Flow on Glucose Uptake in Adipose Tissue

The impact of blood flow on glucose uptake is dependent on insulin. The same equations for adipose tissue are used for muscle glucose uptake (Equations 6-8).

(19) $$\begin{array}{l} \frac{d}{dt}\left(INS_{f_{bf}}\right)=C2_{bf}\cdot \left(I-I_{b}\right)-C1_{bf}\cdot INS_{f_{bf}} \end{array}$$

where *I* is insulin in plasma and *I*_*b*_ is the basal insulin level, and where *C*1_*bf*_ and *C*2_*bf*_ are rate constants.

Second, to calculate the impact of blood flow, we need to have an expression for how the blood flow is calculated. In this study, we only look at blood in controls, and in presence of Bradykinin, which increases the blood flow. This increase is also dependent on insulin. This control of blood flow, denoted *bf*_*f*_ is given by

(20) $$\begin{array}{l} {bf}_{f}=\left(be+kbf\cdot \left(INS_{f_{bf}}+INS_{offset}\right)\right)\cdot bradykinin \end{array}$$

where *be* describes the direct effect of Bradykinin on blood flow; where *kbf* describes the combined effect of insulin and Bradykinin, and where *INS*_offset_ is a small offset introduced to make insulin concentrations positive (same as in Nyman et al., 2011). The value of *bradykinin* is 1 in the absence of Bradykinin, and 2.2 in the presence of Bradykinin.

Finally, the blood flow and insulin are combined to impact the glucose uptake via the following expression for *bfe*_*f*_

(21) $$\begin{array}{l} {bfe}_{f}=\left({bf}_{f}-{bf}_{b}\right)\cdot \left(INS_{f_{bf}}-INS_{b}\right)\cdot p_{bf} \end{array}$$

where *bf*_*b*_ is the basal blood flow, where *p*_*bf*_ is a parameter, and where *INS*_*b*_ is the basal insulin level in adipose tissue.

These are all the equations that have been changed in the current version of the model. The full set of ODEs from the final model, including the original simulation files, are found in the Supplementary Material. The equations are also visualized in the interaction graph in Supplementary Figure 1.

### Model Simulation

The initial values were obtained through steady state simulation, except for the initial values for the Dalla Man submodule, which were kept the same as in Dalla Man et al. (2007). All Initial values used in the simulations can be found in Supplementary Table 4.

### Total Glucose Uptake

**Figure 2:**
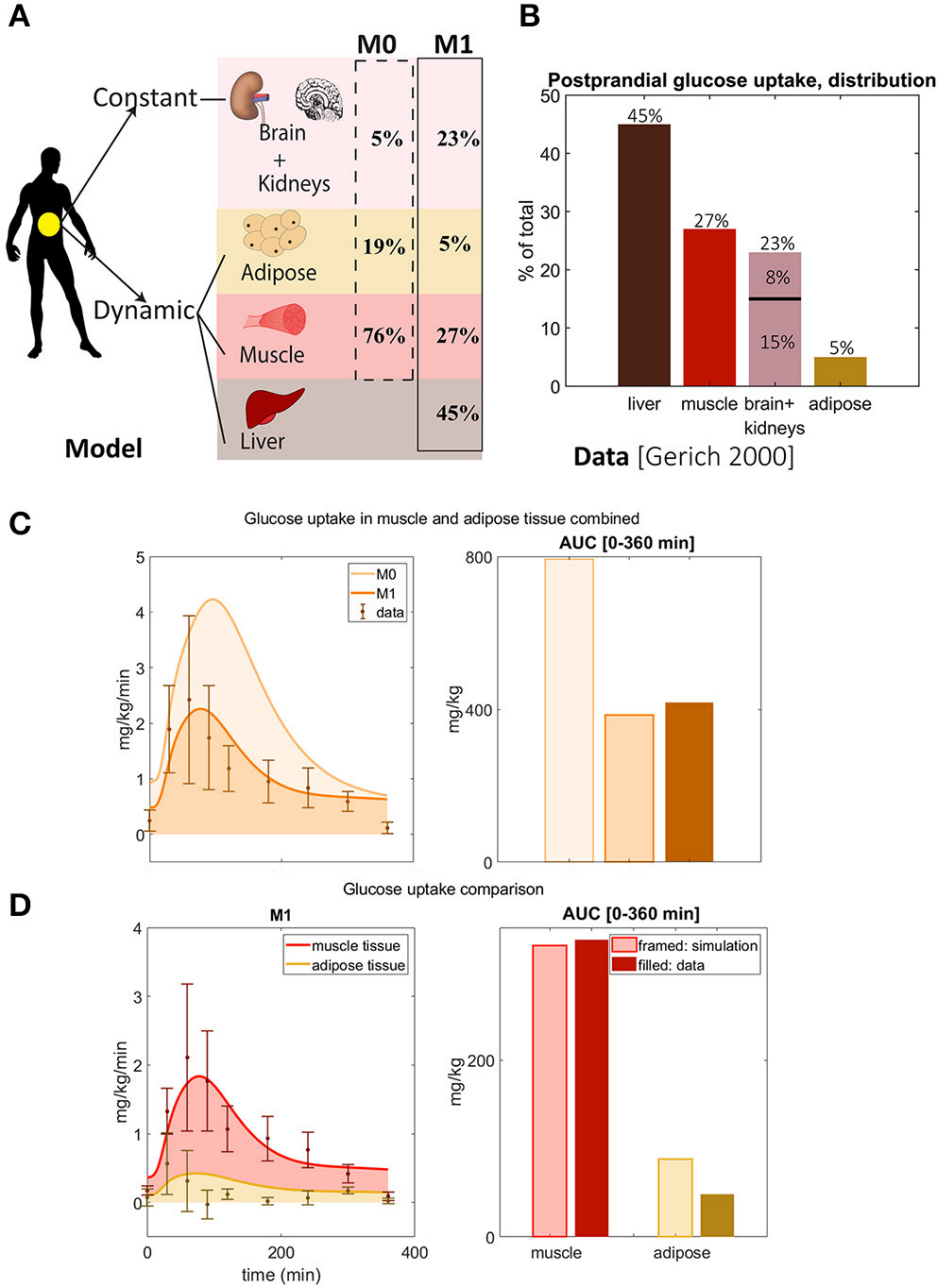
Updated distribution of glucose uptake among tissues. **(A)** In model M1, liver is added, the amount of glucose utilization in muscle and adipose is reduced, and the uptake that is constant during a meal of other tissues is increased compared to the original M0 model. Note that there is no glucose uptake in the liver in M0. **(B)** Glucose distribution among organs observed in data from Gerich (2000). Note that the data shows that the liver is the largest source of glucose uptake. **(C)** Glucose uptake in muscle and adipose tissue combined for M1 and M0. In M1, parameter *U*_*ii*_ was changed, and parameters *part*_*m*_, *part*_*f*_, and *part*_*l*_ were added and chosen to reflect the data of total glucose uptake in **(B)**. The area under the curve for M0 is higher than seen in data from Frayn et al. (1993) and Coppack et al. (1996), and M0 is thus rejected. M1 correctly predicts total glucose uptake based on the independent data from Frayn et al. (1993) and Coppack et al. (1996). **(D)** Comparison between model M1's predictions of adipose and muscle glucose uptake with new data not used for parameter estimation (Frayn et al., 1993; Coppack et al., 1996). M1 correctly predicts independent data for muscle tissue, while it slightly over-predicts independent data for adipose tissue due to timing issue.

**Figure 3:**
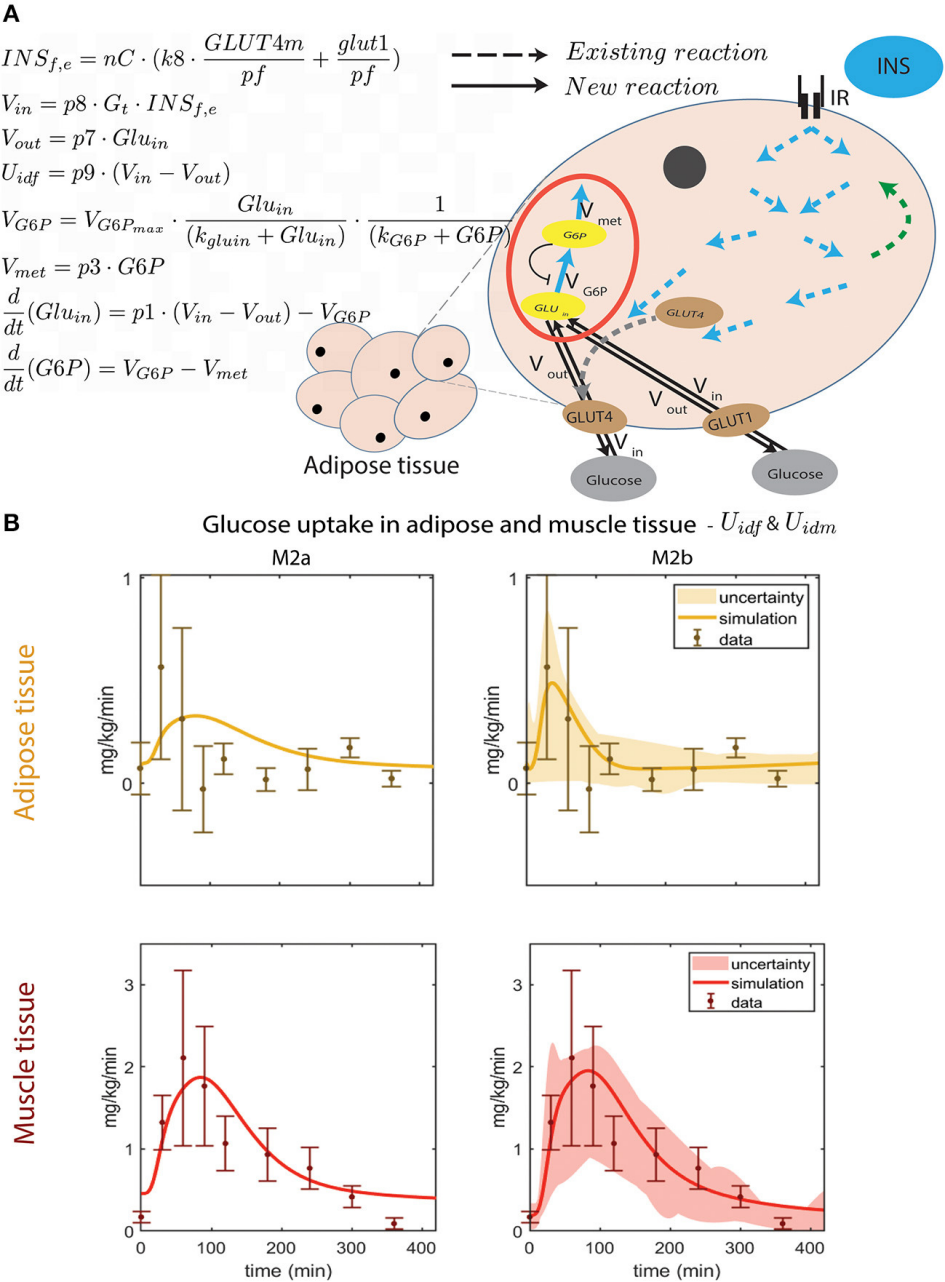
Improved dynamic behavior of adipose tissue glucose uptake by improved intracellular module. **(A)** Illustration of the new intracellular adipose tissue module and ODE equations added to M2b. The flow of glucose in to the cell, *V*_*in*_, is dependent on the amount of glucose in interstitium (*G*_*t*_) and inside the cell (*Glu*_*in*_), and the amount of *GLUT*4*m* and *GLUT*1 membrane glucose transporters through *INS*_*f,e*_. The out flow, *V*_*out*_, is only dependent on *Glu*_*in*_, which in turn depends on, *V*_*in*_, *V*_*out*_, and the phosphorylation of glucose into G6P (*V*_*G*6*P*_). The rate of phosphorylation to *G*6*P* is only dependent on *V*_*G*6*P*_ and the usage of *G*6*P* in metabolism (*V*_*met*_). These equations are detailed in Methods, Equations (11–18). In M2b, the parameters introduced in these equations are optimized together with *V*_*m*_, *V*_*mx*_, *K*_*m*_, *V*_*l*_, *V*_*l*_, *K*_*l*_, *U*_*ii*_, *part*_*m*_, *part*_*f*_, and *part*_*l*_. For all optimized parameters in M2b with a description and their values, see Supplementary Table 4. The equations of the intracellular module that already existed in the original multi-level model (i.e., those of M0, represented by dotted lines for reactions) are kept unchanged in all models presented herein. They are represented by the dotted arrows and describe the insulin induced intracellular signaling, resulting in glucose uptake in the cell. Specifically, they represent the phosphorlyation of intracellular proteins. For example, the two blue arrows pointing out from IR (the insulin receptor) represent the IR-induced phosphorylation of IRS1. For details on this part of the intracellular adipocyte module, see Equations (23–50) in supplementary Figure 1, and (Nyman et al., 2011). **(B)** Timing comparison between uptake seen in data and the two models: M2a without phosphorylation, and M2b with glucose phosphorylation. In M2b, the peak comes earlier and the quantity of glucose taken up is closer to data than in M2a.

The total glucose uptake was approximated with the area under the curve (AUC) of the simulation curves for the 6 h that the dynamic data (Figures 2C,D, 3, 6) was measured for. Specifically, it was calculated using the Matlab function trapz.

### Parameter Estimation

Parameter values for existing models are used from Brännmark et al. (2013). The agreement between model simulations and experimental data is used to estimate values for new model parameters. This agreement is done by minimizing the distance between estimation data, denoted *y*, and corresponding simulated data for parameter *p*, which is denoted ŷ(*p*). In our case, the estimation data consists of uptake rates of glucose into the adipose tissue and muscle, which are denoted *U*_*idf*_ and *U*_*idm*_, respectively. The cost function used is the conventional weight least square, i.e.,

(22) $$\begin{array}{l} v\left(p\right)=\overset{N}{\underset{i=1}{\sum }}{\left(\frac{y_{i}-\hat{y_{i}}\left(p\right)}{SEM_{i}\left(t\right)}\right)}^{2} \end{array}$$

where the subscript *i* denotes the data point, where *N* denotes the number of data points, and where SEM denotes the standard error of the mean for the data uncertainty (Cedersund and Roll, 2009).

We use a χ^2^-test to evaluate the agreement between model simulations and data. To be more specific, we use the inverse of the cumulative χ^2^ distribution function for setting a threshold, and then compare the cost function *v*(*p*) with a threshold.

Fourteen parameters present in the new equations presented herein are optimized. These parameters are *k*_1_, *k*_2_, *K*_*m*_, *V*_*m*_, *V*_*mx*_, *K*_*m*_, *V*_*l*_, *V*_*lx*_, *K*_*l*_, *p*_1_, *p*_2_, *p*_3_, *p*_4_, *k*_*gluin*_, *k*_*G*6*P*_, *V*_*G*6*Pmax*_, and *U*_*ii*_. For detailed description of these parameters, see description of equations above or Supplementary Table 3. All other parameters are fixed and set to values used in Nyman et al. (2011), and these values are found in Supplementary Tables 1, 2. We exploited the modular structure of the model by fitting the parameters in the adipose tissue sub-model separately. In this sub-problem parameter estimation, the input to the adipose tissue sub-model, *G*_*t*_, was considered as a fixed input signal. In the final simulation with the multi-level model, all aspects of the model are simulated at the same time.

The parameter values were estimated using the particleswarm and simulannealbnd algorithms in Matlab R2020b, the former run before the other. The optimization was restarted multiple times, and partially run in parallel at the local node of the Swedish national supercomputing centre (NSC). The parameter estimation was allowed to freely find the best possible combinations of parameter values within boundaries.

Apart from the formal optimization described above, some additional *ad-hoc* requirements were added to the parameter estimation. Specifically, to get a good estimate of the proportions of glucose taken up by the different tissues, a term that adds slightly increasing punishment for having a total uptake of glucose in liver higher than 50% or lower than 40% of total glucose uptake in all organs. The total glucose uptake of other organs except adipose, muscle and liver tissue (*U*_*ii*_) was punished in the same way for values higher than 30 % and lower than 18 % total glucose uptake of all organs. Furthermore, the simple fitting to the subdivision of glucose uptake between muscle, fat and adipose tissue, as well as the fitting to the impact of blood flow on glucose uptake was done by hand. Finally, a representative simulation was chosen for the comparison to the data uncertainties for total glucose uptake from Dalla Man et al. (2007) (Figure 5).

### Uncertainty Estimation

The uncertainty of the model was estimated using step 1 in Cedersund (2012). This step consists of a re-utilization of the previous optimization step: one then saves all parameter sets found by the optimization algorithm, which are acceptable according to a χ^2^-test (see section above). To increase the variation found using this method, a wide variety of starting guesses was performed, when initiating the optimization. After this set of acceptable parameter values were found, these values were used as a basis for simulations, and the maximal and minimal values of these simulations were plotted to get an estimate of the uncertainty of the model.

### Code and Data Availability

We used MATLAB R2020b (MathWorks, Natick, MA) and the IQM toolbox (IntiQuan GmbH, Basel, Switzerland) for modeling. The experimental data as well as the complete code for data analysis and modeling are available at https://gitlab.liu.se/ISBgroup/projects/updated-multi-level, https://zenodo.org/record/4524626#.YCME9OhKguU, and at https://codeocean.com (doi: 10.24433/CO.9800652.v2).

### Experimental and Clinical Data

No new data were collected in this study. We therefore refer to the methods sections in the original articles (Frayn et al., 1993; Coppack et al., 1996; Gerich, 2000; Iozzo et al., 2012; Moore et al., 2012; Brännmark et al., 2013) for the corresponding experimental methods.

## Results

### Distribution of Postprandial Glucose Uptake Between Adipose, Muscle, and Liver (Q1)

We present the model development as a series of smaller improvements, with associated rejections of the previous model without that improvement, to demonstrate exactly why each model improvement has been made. The first improvement made to the original model (Nyman et al., 2011), referred to as M0, was to update the redistribution of the glucose uptake among the different tissues (Figure 2A). The liver stands for almost half of the total postprandial glucose uptake (Figure 2B) (Gerich, 2000), which was not explicitly accounted for in M0 (Figure 2A, dotted line). We therefore adopted the fluxes to fit to the data in Figure 2B. More specifically, the liver was added as a glucose consuming organ, with a high net consumption compared to the other organs. In the updated model, referred to as M1 (Table 1), the liver is set to take up 45% of the total postprandial glucose uptake (Figures 2A,B), while adipose and muscle uptake were both reduced to approximately 5 and 27%, respectively. Furthermore, the glucose uptake by organs whose uptake is not affected by a meal (e.g., brain and kidneys) was changed to 23%. Note that in Figure 2A, this constant uptake is symbolized by the kidneys and the brain, because those are the most prominent glucose consumers (Gerich, 2000), but that other tissues and organs can be seen as represented in this uptake as well.

**Table 1:** Differences between the herein presented models.

**Legend table for modifications**			
M_0_	Original multi-level model		Rejected by Figure 2
M_1_	Updated glucose distribution among organs Adding equations 3,9,10	Can describe Figure 2	Rejected by Figure 3
M_2*a*_	Updated glucose dynamic behaviors by improving interstitial insulin. Modifying/adding equations 6–8		Rejected by Figure 3
M_2*b*_	Updated glucose dynamic behaviors by redesigning an intracellular model Adding equations 11–18	Can describe Figure 3	Rejected by Figure 4
M_3_	The impact of blood flow and insulin has an effect on adipose tissue glucose uptake Adding equations 19–21	Can describe Figure 4	
M_4_	Combining all updates and reformulating to enable moular simulation	Can describe Figure 5	

*Each modification is implemented based on the previous one, e.g., M2 is implemented based on M1. The final model, M4, combines all new additions made in earlier models. All new equations are described below*.

As a validation of these changes, we compared the resulting model simulations with data from other studies. More specifically, we compared the uptake of glucose in adipose and muscle tissue, as simulated by the two models M0 and M1, with data that measures the uptake in these two organs specifically (Figure 2C). Such measurements are possible using e.g., AV difference data. In Figure 2C, the AUC for M0 of adipose and muscle combined (dashed, light orange) is approximately 2 times bigger than the AUC of the data (solid, brown) in (Frayn et al., 1993; Coppack et al., 1996). Note that the model has not been fitted to this data. This is clearly beyond the experimental uncertainty, and M0 is therefore rejected by a χ^2^ test $\left[V\left(\theta \right)=77.7>16.9=\chi_{\text{cum,inv}}^{2}\left(9,0.05\right)\right]$. In contrast, M1 has approximately the same AUC as the data, and its simulations lies within the experimental uncertainty for most data points. Therefore, the time series is not rejected by the test based on these independent data $\left[V\left(\theta \right)=5.36<16.9=\chi_{\text{cum,inv}}^{2}\left(9,0.05\right)\right]$. The two data points with the narrowest uncertainties were estimated to have the mean data uncertainty for both the test of M0 and M1. For these reasons, we reject M0, in favor of the new model M1.

A more detailed check of the quality of the updated model M1 is obtained by looking at the muscle and adipose tissue glucose uptake one by one (Figure 2D). For muscle (red), both the time-dynamics (left) and AUC (right) agrees between simulations (light red) and independent data (dark red). This visual observation is supported by a χ^2^ test $\left[V\left(\theta \right)=4.9<16.9=\chi_{\text{cum,inv}}^{2}\left(9,0.05\right)\right]$. In contrast, the adipose tissue shows a reasonable agreement with data, but it is not quantitatively acceptable according to a χ^2^ test $\left[V\left(\theta \right)=29.5>16.9=\chi_{\text{cum,inv}}^{2}\left(9,0.05\right)\right]$. Note also the discrepancy between the steady-state value of the simulation and the data in both 2C and D—the data starts and ends at almost 0 mg/kg/min, while the simulations end at values closer to 1. This discrepancy is due to the model not being fitted to this data. Looking closer at the time-series reveals that the value at the maximal uptake is fine, but that the problems lies in the fact that the dynamics of the uptake in muscle and adipose tissue are different, and that this is not captured in the model.

### Difference in Time-Resolved Glucose Uptake in Adipose and Muscle Tissue (Q2)

Since the timing and agreement with dynamic glucose uptake in the muscle tissue is fine already in the model M1, this model was kept essentially intact. However, one minor modification that effects muscle uptake was introduced. In the previous model (M1), the rate constant of insulin transport into the interstitium (*V*_1_) is assumed to be the same (*k*_1_) as for the rate of the subsequent degradation of insulin (*V*_2_). Since there is no reason for these values to be the same, we updated the model to give these two reaction rates their own rate constants (*k*1, and *k*_2_, respectively). We refitted both parameters together with the other new parameters (introduced below) to the data, and the resulting model is referred to as M2a.

The developments for the adipose tissue glucose uptake needed to be more elaborate, and are available in Figure 3: the new model structure is depicted in Figure 3A and comparison with data is included in Figure 3B. As can be seen, the same difference as was introduced for muscle, M2a, yields a poor agreement with data for the adipose tissue, since the peak is too late. The main problem is that the glucose uptake in the adipose tissue has gone down to baseline levels already after around 100 min, when insulin levels still are high (Dalla Man et al., 2007) (Figure 6). Therefore, since the glucose uptake in the current model cannot go down before insulin goes down, an additional mechanism is needed. One such possible mechanism is the fact that the hexokinase reaction has a product inhibition (May and Mikulecky, 1983). This leads to two new states in the next version of the model (M2b, Figure 3A, red circle): intracellular glucose *GLU*_*in*_ and phosphorylated glucose, G6P. As seen, there is an inhibition from G6P to the rate of phosphorylation of *GLU*_*in*_.

This modification allows for the following chain-of-events. When glucose uptake begins, the amount of intracellular glucose starts to build up, which is then phosphorylated into G6P. When the G6P reaches saturation levels, G6P inhibits the phosphorylation process from intracellular glucose, which leads to increasing intracellular glucose levels. Since the net glucose uptake is driven by the gradient across the cell membrane, this increase in intracellular glucose will decrease the glucose uptake, even though insulin levels still might be high. The resulting simulations of glucose uptake in muscle and adipose tissue (Figure 3B, right), agrees with the data both according to a visual check, and according to a χ^2^ test $\left[V\left(\theta \right)=24.5<28.9=\chi_{\text{cum,inv}}^{2}\left(18,0.05\right)\right]$. Note also that the steady-state values of the simulations is closer to that of the data.

### Improvements in the Intracellular Adipose Tissue Model: Glucose Metabolism and Blood Flow Effects (Q3)

**Figure 4:**
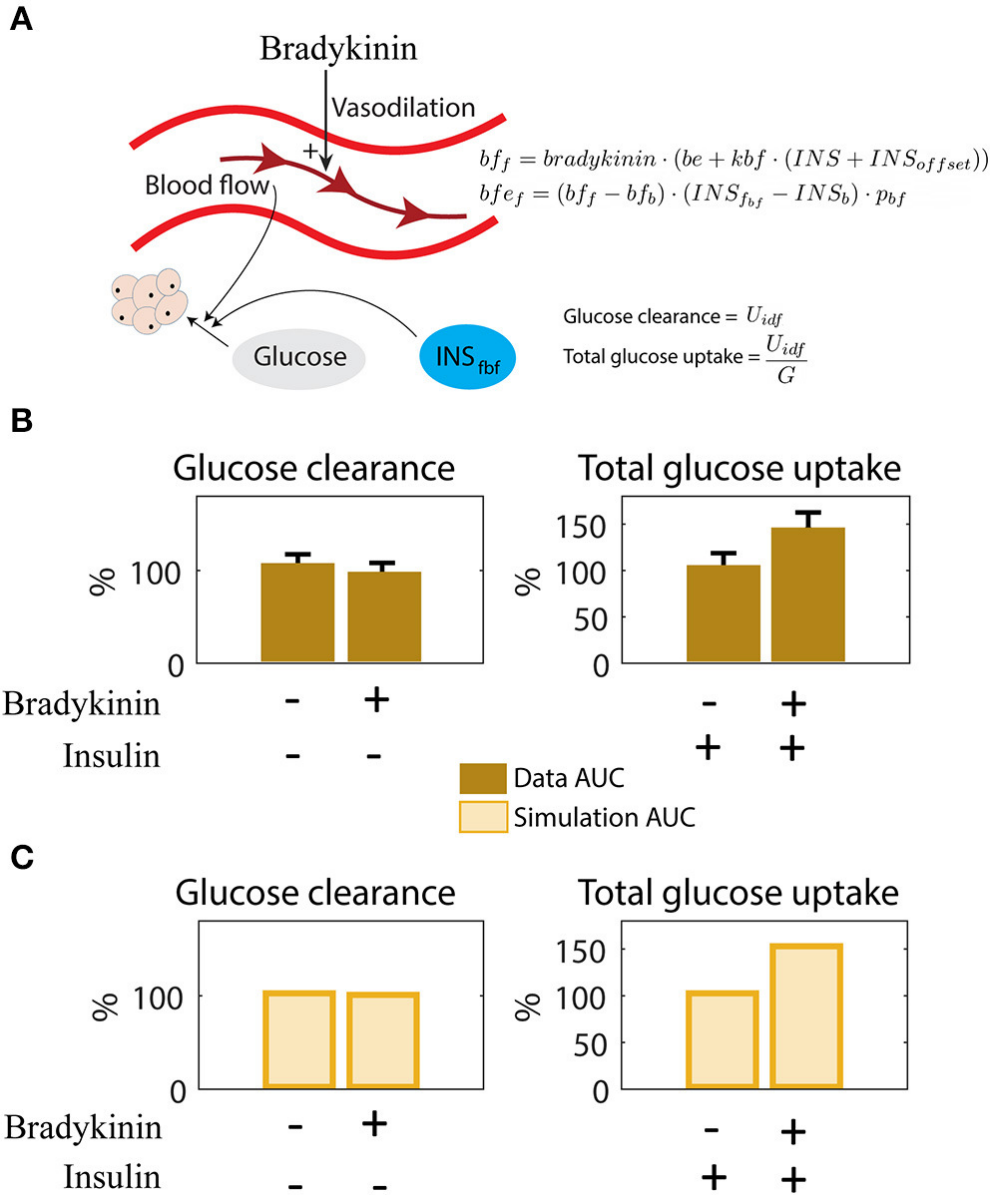
Interaction between blood flow and insulin on adipose glucose uptake. **(A)** Illustration of blood flow and insulins effect on adipose tissue glucose uptake. Bradykinin increases the blood flow. The new equations for blood flow (*bf*), dependent on bradykinin (*bradykinin*), and blood flow effect (*bfe*_*f*_), dependent on blood flow dependent insulin in fat tissue (*INS*_*fbf*_). The parameters changed/added here are *bf*_*b*_, *bradykinin*, *be*, *p*_*bf*_, and *INS*_*b*_. See Supplementary Table 4 for description and value. **(B)** Behavior seen in data as response to insulin and Bradykinin. Insulin alone has a relatively small effect on glucose clearance, but increases glucose uptake significantly when combined with Bradykinin (Iozzo et al., 2012). **(C)** The same behavior as in **(B)** (Iozzo et al., 2012) can be simulated with the model. Adding Bradykinin is simulated by increasing the value of *bradykinin*, and adding insulin infusion is simulated by increasing the value of *INS*_offset_ from 0.

The final improvement made was the addition of the impact of blood flow on insulin-stimulated glucose uptake in the adipose tissue. This interaction was hypothesized in Iozzo et al., where they looked at the effect of blood flow and insulin, separately and combined, on glucose uptake in adipose tissue (Iozzo et al., 2012) (Figures 4A,B). Increased blood flow was achieved with the drug Bradykinin. In these experiments, Iozzo et al. observed that glucose clearance was not significantly changed when only adding Bradykinin (Figure 4B, left). In contrast, when combining both Bradykinin and insulin, the glucose uptake is increased compared with only adding insulin (Figure 4B, right). The same behavior is produced by the model in Figure 4C, where the glucose uptake only increases when both Bradykinin and insulin is present. The parameter *bradykinin* was changed from 1 to 3,500 (estimated by hand) to represent the addition of Bradykinin, and the parameter *INS*_offset_ is changed from 0 to 7 to represent insulin infusion (Figure 4A). This behavior also agrees with data according to according to a χ^2^ test [$V\left(\theta \right)=0.26<3.8=\chi_{\text{cum,inv}}^{2}\left(2,0.05\right)$, where the degrees of freedom have been compensated for with the number of new parameters, 4-2 = 2]. The updated model is referred to as M3, and as for the other model additions, the new equations are shortly depicted in the figure (here Figure 4A), and described in detail in Materials and Methods and Supplementary Files.

### The Final Model (Q4)

**Figure 5:**
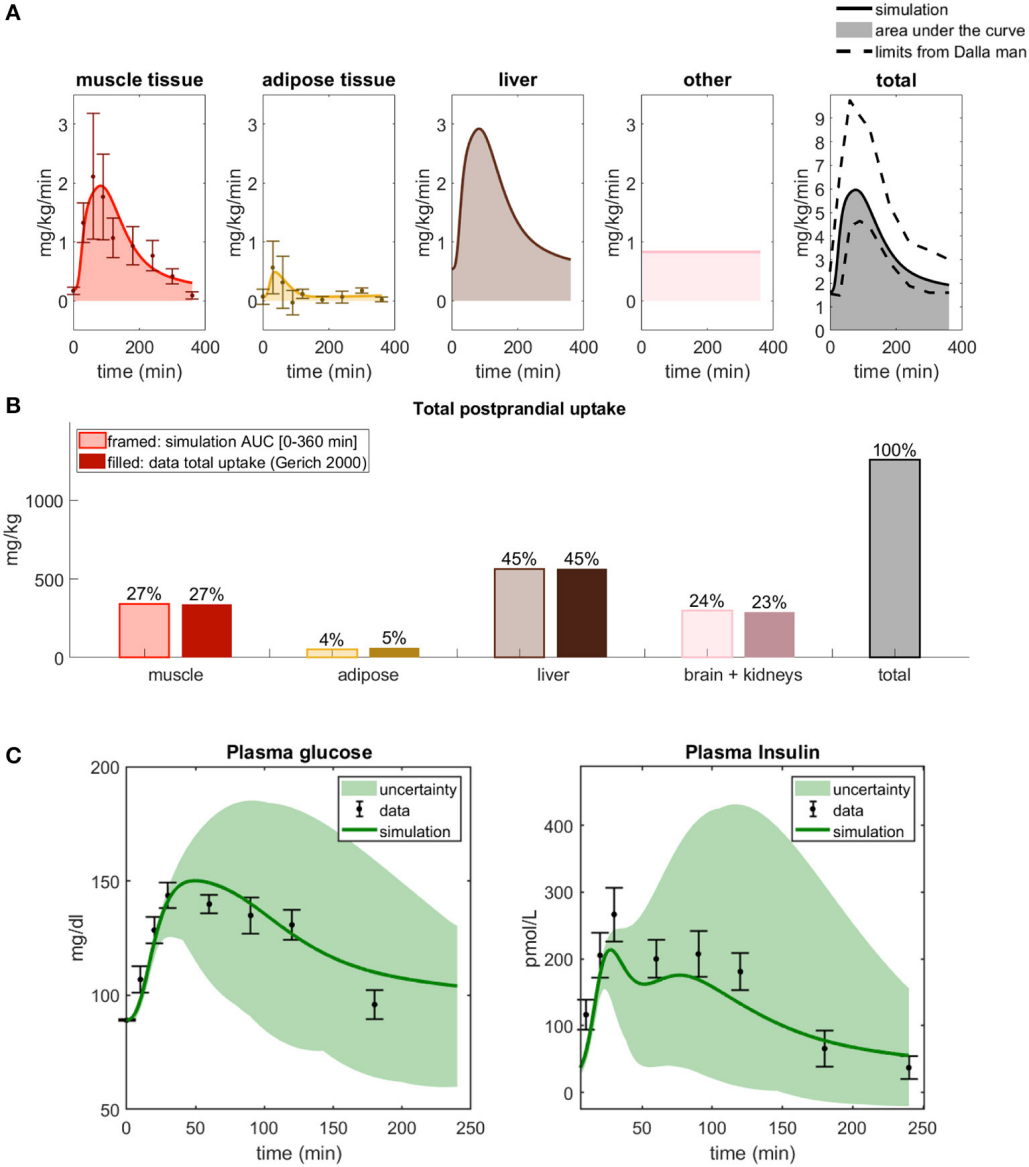
The behavior of the final updated multi-level model. **(A)** Simulations of glucose uptake in all organs and tissues and time-series for the data used to fit the model (Coppack et al., 1996). The total glucose uptake is within the bounds presented in Dalla Man et al. (2007). **(B)** Total glucose uptake for all organs, simulated by the final model and from the data used to fit the model (Coppack et al., 1996). **(C)** Simulations of plasma glucose and insulin compared with independent data from Adams et al. (2018). The green line represents the parameter set with the best fit to validation data.

Finally, we consider the performance of the resulting final multi-level model, in relation to all of the data that has been generated over the years. The final model can fit to dynamic data of postprandial glucose uptake in both adipose and muscle tissue (Figure 5A, same data as in Figure 2D, from Coppack et al., 1996). The same figure displays predictions of dynamic uptake in the liver (for which the same type of AV difference data is non-existent), and for the tissues with a constant demand of glucose (such as the brain). Finally, the right-most sub-figure in Figure 5A shows that the model agrees well with the total dynamic glucose uptake from Dalla Man et al. (2007).

Furthermore, the AUCs for the 6h simulations of the different tissues in the final combined model is in line with the corresponding AUC data (Figure 5B), just as they were in step Q1 (Figure 2B). The two left-most bars, for muscle and adipose tissue, are given by the AUC of the corresponding time-series in Figure 5A (cf Figure 2D), and the liver and brain/kidney uptake are the same as in Figure 2B. To further test the resulting final model, we have compared it with a new data set from another paper, not used for model training (Adams et al., 2018). As can be seen, the model agrees with the data [$V\left(\theta \right)=29.8<30.6=\chi_{\text{cum,inv}}^{2}\left(15,0.01\right)$]. (The test and simulated line in Figure 5C concerns the found parameter set with the best fit to validation data.)

**Figure 6:**
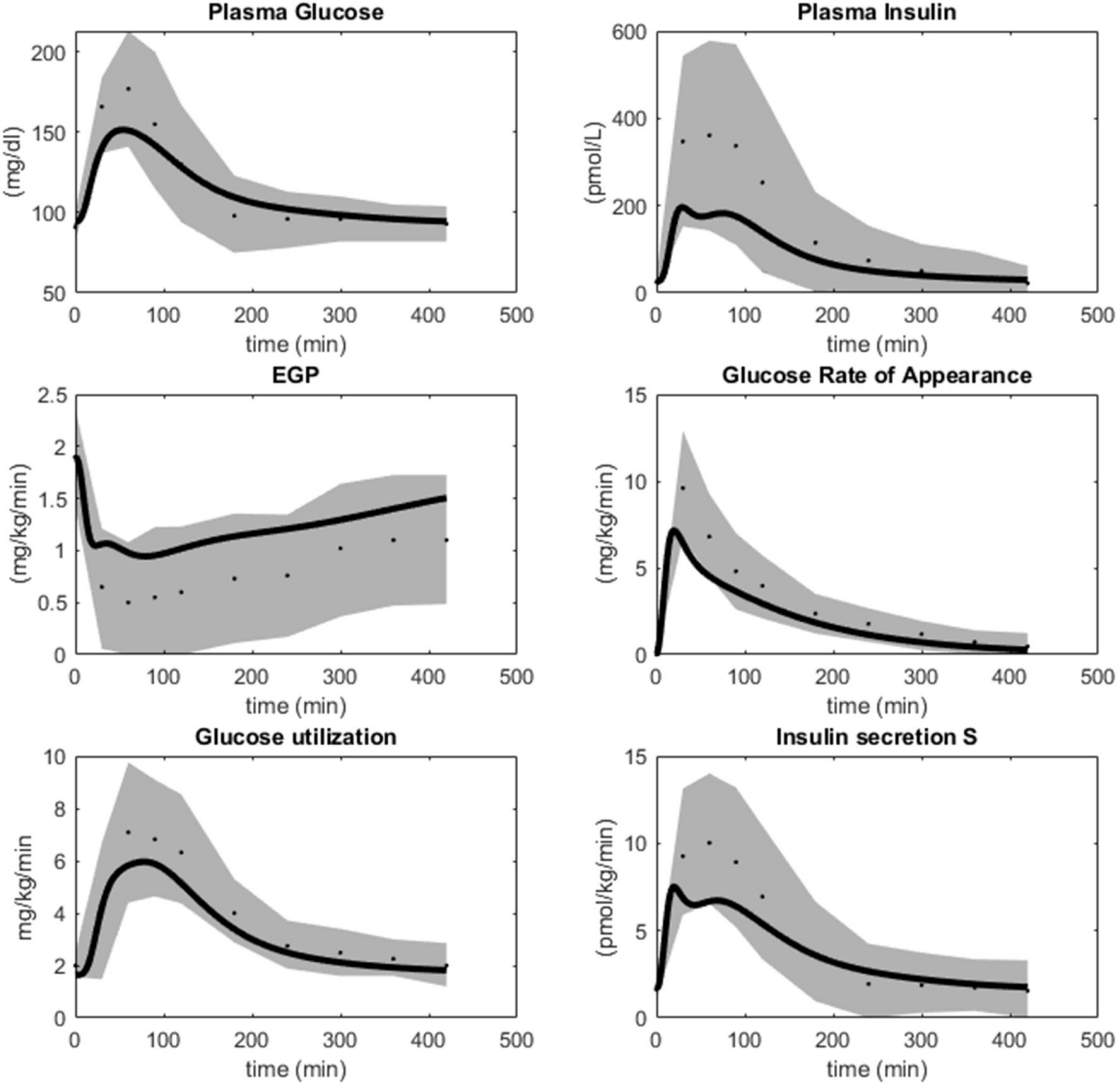
Simulations of M4 (lines) in comparison with data (dots) and uncertainties (gray areas) from Dalla Man et al. (2007). M4 simulations are within the data uncertainties established in Dalla Man et al. (2007).

The final model is also in agreement with data previously used in the model development. The agreement with the most important such data sets are re-plotted in Figure 6 (Dalla Man et al., 2007), which describes meal responses for the following variables: Plasma Glucose, Plasma insulin, Endogenous Glucose Production, Glucose Rate of Appearance from the intestines, Glucose uptake or utilization, and insulin secretion. As can be seen, the model simulations (lines) are within the experimental uncertainty (gray area) for all these time curves (agreements between simulation and data are similar as in Dalla Man et al., 2007).

**Figure 7:**
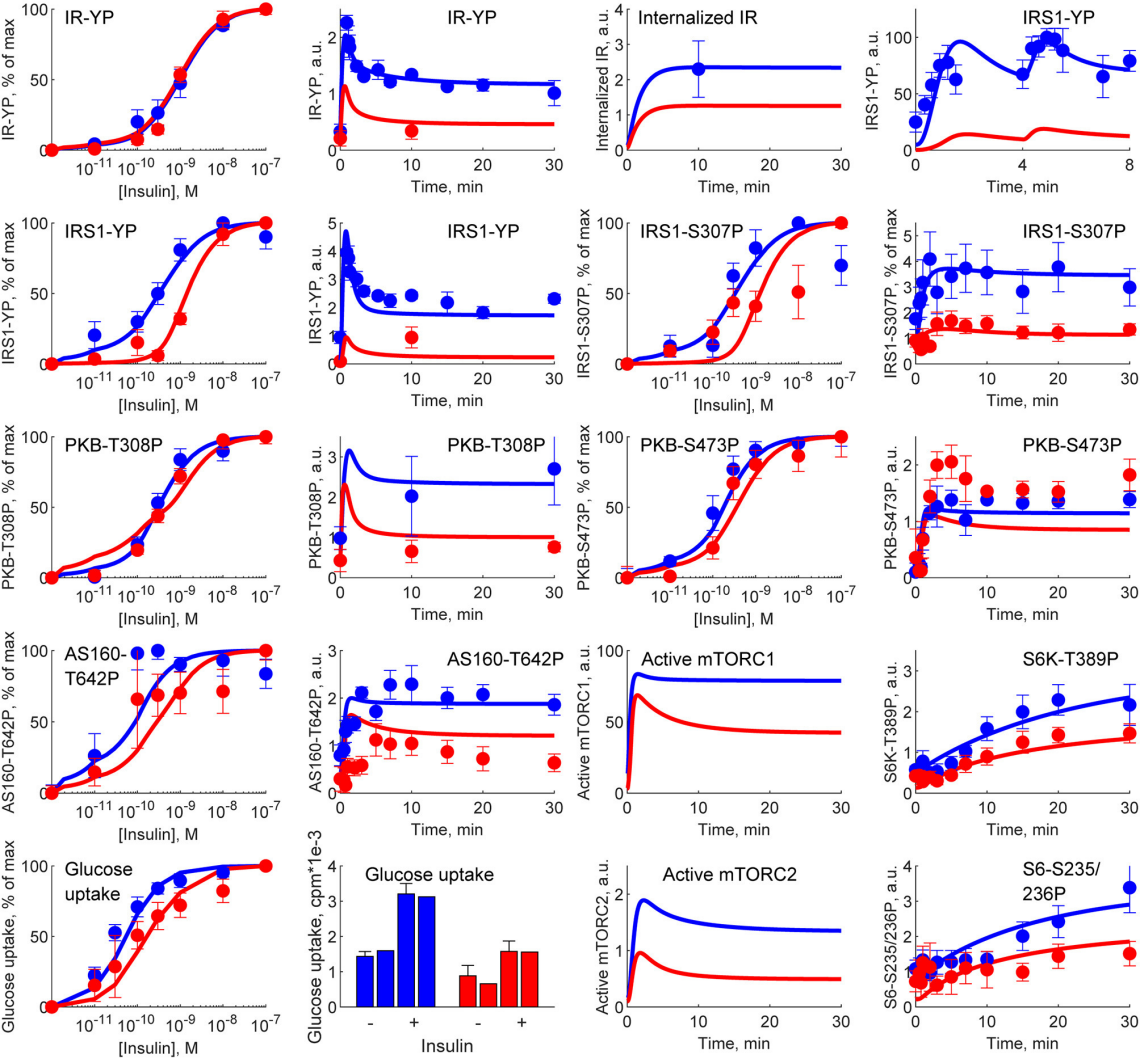
Simulations of M4 (lines) in comparison with experimental data (error bars) from Brännmark et al. (2013). M4 can describe data for intracellular insulin signaling in adipocytes, both normally (blue) and in T2D (red). IR, insulin receptor; IRS1, insulin receptor substrate-1; PKB, protein kinase-B; AS160, Akt-substrate 160; S6K1, Ribosomal protein S6 kinase beta-1; S6, Ribosomal protein S6; YP, tyrosine phosphorylation; SP,serine phosphorylations (on sites 235/236, 307, 473); TP, threonine phosphorylation (on site 308).

Similarly, because of the hierarchical way that the multi-level model is constructed, it also still agrees with all of the intracellular signaling data, which we have collected over the years (Brännmark et al., 2013). The most important such data is depicted in Figure 7. These data (error bars) describe time-series and dose-response curves in response to insulin for a number of intracellular proteins: the insulin receptor (IR), the insulin receptor substrate-1 (IRS1), protein kinase-B (PKB), Akt-substrate 160 (AS160), Ribosomal protein S6 kinase beta-1 (S6K1), Ribosomal protein S6 (S6), as well as cellular glucose uptake. The model simulations (lines) are in agreement with both data from non-diabetic and lean controls (blue), and from obese people with type 2 diabetes (red), with changes only in a few key parameters (for more details, see Brännmark et al., 2013). Similar agreements for additional proteins—such as extracellular signal-regulated kinases (Erk1), ETS Like-1 protein Elk-1 (Elk1), Forkhead box protein O1 (FOXO1), etc—is equally possible to obtain by replacing the intracellular part of the model with those in Nyman et al. (2014) and Rajan et al. (2016).

## Discussion

Glucose homeostasis is a complex multi-organ and multi-level system, which requires multi-level mathematical modeling for a full understanding. We have herein improved an existing such model (Nyman et al., 2011) for glucose fluxes in the circulation, linked to intracellular pathways in adipocytes, in response to a meal. Specifically, we have (Q1) made a new subdivision of glucose uptake between all relevant organs, to provide more reliable proportions and to include uptake in the liver (Figure 2); (Q2) improved the elimination of interstitial insulin to be tissue-specific, and included intracellular metabolism of glucose inside adipocytes, to capture an earlier peak in the glucose uptake in adipocytes compared to the corresponding peak in plasma insulin (Figure 3); and (Q3) accounted for the impact of blood flow on glucose uptake (Figure 4). The final combined model (Q4) can fit to all of the new data for glucose uptake in all organs (Figure 5), as well as to all previous data, such as the postprandial glucose and insulin fluxes and concentrations in Dalla Man et al. (2007) (Figure 6), and the intracellular data in Brännmark et al. (2013) (Figure 7). To the best of our knowledge, this is the most comprehensive description of such a wide variety of data for glucose homeostasis in humans, and we hope that it will become a useful resource also for integration of future data.

One of the main contributions in this work is the addition of glucose uptake in the liver (Q1). This addition is important because the liver is the organ that takes up the most postprandial glucose: approximately 45% (Figure 2). The previously published model did not include glucose uptake in the liver (Figure 2A), which is problematic since data shows that it is the biggest source of glucose clearance (Figure 2B). Apart from this, the liver has a unique function in glucose homeostasis, since the liver and the kidneys are the only organs that can produce glucose from other metabolites. These two functions, glucose uptake and EGP, are now modeled as separate processes. In other words, the liver can both produce and take up glucose at the same time. While there may be situations when only the net uptake/release is important, there are also situations when one can experimentally resolve the two fluxes. For instance, when labeled metabolites have been ingested, one can see the rate by which these are converted to glucose and secreted, even in postprandial conditions, when the net effect of glucose transport is into the cell. There exist other models where these kinds of data have been used to model the two liver functions separately, such as in (Knig et al., 2012). Such data have previously also been used to train the EGP fluxes of our model (Figure 6) (Dalla Man et al., 2007), and we have now added corresponding data for glucose uptake (Figure 5B). Note that this model is only fitted to the data in Figure 2B, and that the agreements seen in Figures 2C,D serve as a simple validation of this part of the model. With this said, it should be emphasized that both the muscle and the new liver module are highly simplified. Only the muscle and adipose modules have been tested with respect to dynamic uptake data, and only the adipose module with an intracellular signaling part, based on detailed intracellular data, resolving the complicated intracellular metabolic fluxes. A natural next addition to the model would be that of other detailed cell modules, using similar data for other cell types. Dynamic data of glucose uptake in other tissues (summarized as “other” in Figure 5A) would preferably also be used to develop the model to include the particulars of those processes. The same goes for the effect of blood flow on glucose uptake, which is only detailed for adipose tissue as of now (Figure 4). There is also a significant difference in the amount of data used for the adipocyte module (> 30 time courses) and that used for the systemic glucose homeostasis (2 time courses). These limitations are present primarily because such data are rare or non-existent.

At the heart of resolving both Q1 and Q2 lies measurements of glucose fluxes, which have been measured in a variety of ways. The glucose fluxes from Dalla Man et al. (2007) was based on a triple tracer protocol, which allows for the simultaneous calculation of plasma glucose, EGP, glucose rate of appearance, and glucose utilization (Figure 6). These data are based on advanced calculations, which in turn are based on various assumptions and mathematical models developed within the field of tracer based measurements (Wolfe et al., 2005). These particular assumptions are not necessary in the organ specific glucose utilization curves, available e.g., for muscle and adipose tissue (Figures 2C,D). These data are based on an AV difference-based protocol, which samples in both an artery and veins that have past through either muscle or adipose tissue, and by looking at the difference between the ongoing and the outgoing blood (Coppack et al., 1996). This is a more direct way of measuring how each organ contributes to the glucose disappearance from the blood. Nevertheless, also AV-difference data does not measure glucose uptake in the primary cells, myocytes, and adipocytes, respectively. This means that the quick decline in glucose uptake in adipose tissue (Figure 3B) could in fact be the result of a quick equilibrium between interstitial and capillary glucose concentration. One could possibly develop an alternative model based on that equilibration-based assumption, to explain the quick decline of the glucose uptake in the adipose tissue, either as a replacement or as a complement to the herein implemented mechanism based on product inhibition (Figure 3A). Note that even though some data used have a relatively high uncertainty, these data are still enough to reject the models without the new modifications. Finally, the fact that the model is based on three different types of measurements of glucose uptake (cellular *in vitro*, tracer-based, and AV-difference based), and can describe all of these types of data simultaneously, is a reason why a relatively simple validation, such as that in Figures 2C,D, still is of value.

The final question addressed herein (Q3) concerns the impact of blood flow on glucose uptake, which is highly simplified because the real relationship is a bidirectional one. The data in Figure 4B shows that glucose uptake is increased by increased blood flow, at least in cases when insulin is present. This relationship is captured in the final model. However, that model can only describe situations where the blood flow is altered in a way that is not connected to the metabolic response, such as when adding Bradykinin (Figure 4B). In other words, the model cannot describe meal-induced blood flow changes and its associated impact on glucose uptake. The development of a model for blood-flow regulation during e.g., meal-responses is an important task for future modeling works. Another weakness regarding the blood flow part of the model concerns the lack of validation. The model is only fitted to the data in Figure 5B. In the analysis, we compensate for that by reducing the degrees of freedom from the number of data points (4) to the number of data points minus the number of parameters (4-2 = 2). However, one could argue that the two baseline bars should not be counted since they are normalized to be 100%. In such an interpretation, the degrees of freedom are 0, a chi2 test can not be done, and the only possible assessment of the quality of the model s a visual comparison of the differences between Figures 4B,C. For all these reasons, the blood flow part of the model is to be considered as a first step in the development of a model for the blood flow and its function in glucose homeostasis.

The model agrees with independent data saved for validation, both herein and in previous papers describing sub-models, but there are naturally still many limitations remaining. The main new such validation was, as mentioned, done already in Figures 2C,D, which are data the model has not yet been fitted to. The reason why this is the main validation is that this tests the key novel contribution of this paper: the updated sub-division of glucose uptake profiles in the different organs. We also included a final comparison with respect to independent data, when evaluating the final model, which also that passes a chi-square test (Figure 5C). Apart from these two new validations herein, each constituent sub-model developed previously, has already been tested with respect to such independent validation data. For instance, the whole-body Dalla Man model has been used and tested extensively (Man et al., 2014; Visentin et al., 2014, 2015), and is even accepted by the FDA for certain replacement applications (Kovatchev et al., 2009). Similarly, the intracellular insulin signaling model we use here was presented in Brännmark et al. (2013), which e.g., was tested with respect to independent data describing perturbation results for numerous proteins in response to mTORC1 inhibition (Figure 7 in Brännmark et al., 2013). Since then, the model has added additional proteins, such as Erk1, Elk1 (validation in Figures 6, 7 in Nyman et al., 2014), FOXO1 (Rajan et al., 2016, 2018), etc. However, despite these successful tests with respect to independent data, it is not accurate to say that the models are validated as such, and there are many situations they still cannot correctly predict. For instance, the Dalla Man model has problems when changing between two different populations, when different complex meals are ingested which e.g., not only consist of glucose, when moving to starvation and hypoglycemia, in the presence of exercise, etc. Similarly, the insulin signaling part does not describe most of the proteins involved in the insulin signaling network (Humphrey et al., 2013), other stimuli which cross-talk with insulin signaling, such as insulin-like growth factor (De Meyts, 1994), catecholamines (Krieger and Landsberg, 1988), etc. In summary, the model has been tested with respect to independent data many times, but it is still expected to have problems with many types of predictions.

Another limitation is that the uncertainty estimation of the parameters was done only using Step 1 in Cedersund, 2012). However, an initial profile likelihood analysis (data not shown) shows that the individual parameters are not identifiable, which means that e.g., a more extensive parameter characterization method such as Markov Chain Monte Carlo methods (MCMC) (Cedersund, 2012) probably would not converge. Note that this parameter unidentifiability does not limit any of the conclusions drawn herein, and that some predictions with the model still may be identifiable, even though the parameters are unidentifiable. Such well-determined predictions are sometimes called core prediction, and this topic is further discussed in (Cedersund, 2012).

The complete regulatory system that makes up whole-body and multi-level glucose homeostasis is a highly complex one, and our new model is just an updated framework to incrementally add pieces of data and knowledge as they become available—and not a finished and complete picture. As outlined already in (Cedersund and Roll, 2009; Nyman et al., 2011), we tackle modeling of the glucose homeostasis in a hierarchical way, with modules that have an internal structure that does not need to be understood, when describing the cross-talk between these modules. The top-level version of these modules are the organs, and it is therefore important that the cross-talk between, and relative role of, each organ is correct. This is the main question raised in this manuscript, regarding the distributions (Q1), timings (Q2), and impact of blood flow (Q3). With this division of labor in place, one can then fill in details regarding the internal and intracellular mechanisms in each organ as they become available. In our model, such details are primarily filled in for the adipose tissue, which is where we ourselves have had an experimental capability to investigate those intracellular details for many years. However, the same type of intracellular details can be added for other intracellular processes in other organs, when such *in vitro* data becomes available, and when such data have been properly analyzed using modeling. Note that such subsequent additions to the model can be done with minimal to no change to the rest of the model, because of the hierarchical structure. Therefore, although our model is relatively complex, it is still developed in a minimal way, where details to the different sub-models only are added if there are data available to support and warrant those additions.

It is important to compare the model presented herein to other similar models in the literature. In the introduction, we mentioned the now classical non-linear mixed effects models describing plasma levels of glucose and insulin (Jauslin et al., 2007; Silber et al., 2007, 2010). These models have since these early publications been used to scale data between pre-clinical data from animals to clinical human data for glucose and insulin concentrations (Alskr et al., 2017), and to describe cross-talk with more long-term processes, such as disease development in mice (Choy et al., 2016) and dynamics of HbA1c (Kjellsson et al., 2013; Mller et al., 2013). Glucose homeostasis-centered models, focusing on the glucose-insulin interplay, lie at the heart of mathematical models developed for type 1 diabetes, e.g., to aid insulin-pumps, and to develop a so-called artifical pancreas (Huang et al., 2012; Fabris and Kovatchev, 2020). Another application of glucose homeostasis models exist for meal response T2D simulator model, developed for pedagogical and motivational purposes (Maas et al., 2015). None of these models have subdivided glucose uptake in the different organs, or included intracellular responses, in multi-level and multi-organ models. There exists one model that does this, developed by Uluseker et al. (2018). This multi-level model is based on a version of the Dalla Man model (Dalla Man et al., 2007) connected with our intracellular adipocyte model (Brännmark et al., 2013), while also including hormonal effects on glucose intake/appetite (leptin, ghrelin) and insulin levels (incretin). However, this model does not compare their whole-body simulations with any data, and does not include the liver as a glucose consuming organ. There are also some other multi-level and multi-scale models for other systems that should be mentioned. One such model is the one developed by Barbiero and Lió (2020). This model combines whole-body dynamics with the function of organs and individual cells, and is able to simulate dynamics in seconds up to several days. The model was used to simulate the cardiovascular and inflammatory effect of both T2D diabetes and COVID-19, using personalized parameters. However, this model has a similar important short-coming: its simulations are not compared with any data. There also exists interconnected models for e.g., heart function, describing the function of cardiac cells up to the integrated behavior of the intact heart (Smith et al., 2009). In addition to leptin and ghrelin, there are other hormonal effects on glucose homeostasis that our model disregards but that other models include, such as that of incretins, glucagon, and epinephrine. Knig et al. (2012) developed a detailed model of the role of the liver in glucose metabolism, that includes glucagon and epinephrine. Grespan et al. developed a model of the beta cell, specifically describing the effect of two incretins (GIP and GLP-1) on insulin secretion (Grespan et al., 2021). Furthermore, the model presented in this work only included intake of glucose, and thus discarded the effects of proteins and fat on the meal response, something that other models do take into account, to some extent. Sips et al. developed a model that integrates fatty acids with glucose metabolism (Sips et al., 2015), but this model needs a triglyceride curve as input, and lacks protein metabolism. Nevertheless, the Sips model is another expansion of the Dalla Man model (Dalla Man et al., 2007) and can thus be merged with the developments herein. Two models that include protein and fat intake from a meal are the ones developed by Hall et al. (2011) and Sarkar et al. (2018). These models are however developed for long term simulations (over several years), and can thus not simulate a meal response. In similarity to the model presented here, the Sarkar model include liver, muscle, and adipose tissue as glucose consuming organs, but in contrast also adds the pancreas as a glucose consuming organ. Furthermore, the Sarkar model disregards the organs taking up a constant amount of glucose (brain and kidneys). In any case, the Sarkar model only describes data for long-term dynamics, and does not describe meal-responses. Another longitudinal model describing glucose dynamics on both short and long-term time-scale is the one developed by Ha and Sherman (2020). This model is, in contrast to the other two longitudinal models mentioned above, multi-scale in that it can look at both changes over years, including the progression toward diabetes in a semi-mechanistic fashion, as well as meal response dynamics happening in the scale of hours and minutes. This model does, however, not include the distribution of glucose among different organs.

In summary, there does not exist any other multi-level model describing glucose meal response, that also separates between the different organs' glucose uptake. In this work, we present such a model, that, due to its modular approach can be easily expanded in different directions. This expansion-possibility is due to both the modular structure, and to the fact that each module can be treated as a separate modeling problem. In other words, as long as the model for each module agrees with the input-output profiles of insulin and glucose, the new model can replace the old model, with little alterations on whole-body dynamics. In the earlier developed model (Nyman et al., 2011), we took this modularity one step further, by replacing the simpler 5-state insulin receptor module with a much more detailed 37-state module for the receptor dynamics, including the possibility for a receptor to bind up to three insulin molecules (Kiselyov et al., 2009). This demonstrates the usefulness of developing a model in modules, so that the right level of details can be included depending on the data/questions you want to analyze.

Finally, since the original publication of our first multi-level model (Nyman et al., 2011), we have built further on this model in several directions, and all of these developments can be re-used also in our new model. We have e.g., expanded the intracellular part to explain a more and more comprehensive picture of the alterations in intracellular signaling that occur in T2D. This has been done by taking adipose tissue biopsies from both healthy and T2D individuals, and characterize their respective insulin signaling. In Brännmark et al. (2013), we presented a first model of how the insulin resistance occurs, and in subsequent works, we have added additional proteins, such as FOXO1 transcription factor (Rajan et al., 2016), insulin control of MAPKs ERK1/2 (Nyman et al., 2014). Because of the modular way that our multi-level model is structured, one can replace the herein used intracellular model with any of these other alternatives. The same expansions can be done also for other organs. We therefore hope that this multi-level model in the future can serve as a hub for connecting data and models together into a useful systems-level understanding.

## Data Availability Statement

The experimental data as well as the complete code for data analysis and modeling are available at: https://gitlab.liu.se/ISBgroup/projects/updated-multi-level; https://zenodo.org/record/4524626#.YCME9OhKguU, and at codeocean.com (doi: 10.24433/CO.9800652.v2).

## Author Contributions

TH: Performed parameter optimization, produced plots for results, and writing manuscript. HL: Developed first version of model and produced parts of figures. EN: Guidence in parameter estimation and writing and proof reading manuscript. GC: Developing research plan, developing hypothesis behind model, and writing and structuring manuscript. All authors contributed to the article and approved the submitted version.

## Conflict of Interest

The authors declare that the research was conducted in the absence of any commercial or financial relationships that could be construed as a potential conflict of interest.

## Abbreviations

T1D: Type 1 diabetes
T2D: Type 2 diabetes
AUC: Area under the curve
EGP: Endogenous glucose production
AV: Arteriovenous
ODEs: Ordinary differential equations.
