# Supplementary material for: An Updated Organ-Based Multi-Level Model for Glucose Homeostasis: Organ Distributions, Timing, and Impact of Blood Flow
Source: Front Physiol. 2021 Jun 1;12:619254. doi: 10.3389/fphys.2021.619254 (PMC8204084; doi:10.3389/fphys.2021.619254)
Supplement: Supplementary file 1 [file Data_Sheet_1.pdf]

An Updated Organ-Based Multi-Level Model for Glucose Homeostasis: Organ Distributions, Timing, and Impact of Blood Flow: Supplementary Material

All the ODEs for the final model M4:

$$\frac{d}{dt}(IR) = -v1a - v1basal + v1r + v1g \tag{1}$$
$$\frac{d}{dt}(IRp) = v1basal + v1c - v1d - v1g \tag{2}$$
$$\frac{d}{dt}(IRins) = v1a - v1c \tag{3}$$
$$\frac{d}{dt}(IRip) = v1d - v1e \tag{4}$$
$$\frac{d}{dt}(IRi) = v1e - v1r \tag{5}$$
$$\frac{d}{dt}(IRS1) = v2b + v2g - v2a - v2basal \tag{6}$$
$$\frac{d}{dt}(IRS1p) = v2a + v2d - v2b - v2c \tag{7}$$
$$\frac{d}{dt}(IRS1p307) = v2c - v2d - v2f \tag{8}$$
$$\frac{d}{dt}(IRS1307) = v2basal + v2f - v2g \tag{9}$$
$$\frac{d}{dt}(X) = v3b - v3a \tag{10}$$
$$\frac{d}{dt}(Xp) = v3a - v3b \tag{11}$$
$$\frac{d}{dt}(PKB) = -v4a + v4b + v4h \tag{12}$$
$$\frac{d}{dt}(PKB308p) = v4a - v4b - v4c \tag{13}$$
$$\frac{d}{dt}(PKB473p) = -v4e + v4f - v4h \tag{14}$$
$$\frac{d}{dt}(PKB308p473p) = v4c + v4e - v4f \tag{15}$$
$$\frac{d}{dt}(mTORC1) = v5b - v5a \tag{16}$$
$$\frac{d}{dt}(mTORC1a) = v5a - v5b \tag{17}$$
$$\frac{d}{dt}(mTORC2) = -v5c + v5d \tag{18}$$
$$\frac{d}{dt}(mTORC2a) = v5c - v5d \tag{19}$$
$$\frac{d}{dt}(AS160) = v6b1 - v6f1 \tag{20}$$
$$\frac{d}{dt}(AS160p) = v6f1 - v6b1 \tag{21}$$
$$\frac{d}{dt}(GLUT4m) = (v7f - v7b) \tag{22}$$
$$\frac{d}{dt}(GLUT4) = -v7f + v7b \tag{23}$$
$$\tag{24}$$

$$\frac{d}{dt}(S6K) = v9b1 - v9f1 \tag{25}$$

$$\frac{d}{dt}(S6Kp) = v9f1 - v9b1 \tag{26}$$

$$\frac{d}{dt}(S6) = v9b2 - v9f2 \tag{27}$$

$$\frac{d}{dt}(S6p) = v9f2 - v9b2 \tag{28}$$

$$\frac{d}{dt}(G_p) = EGP + Ra - E - U_{ii} - k_1 \cdot G_p + k_2 \cdot G_t \tag{29}$$

$$\frac{d}{dt}(G_t) = -U_{id} + k_1 \cdot G_p - k_2 \cdot G_t - U_{idl} \tag{30}$$

$$\frac{d}{dt}(I_l) = (-m_1 \cdot I_l) - m_3 \cdot I_l + m_2 \cdot I_p + S \tag{31}$$

$$\frac{d}{dt}(I_p) = (-m_2 \cdot I_p) - m_4 \cdot I_p + m_1 \cdot I_l \tag{32}$$

$$\frac{d}{dt}(Q_{sto1}) = -k_{gri} \cdot Q_{sto1} \tag{33}$$

$$\frac{d}{dt}(Q_{sto2}) = (-k_{empt} \cdot Q_{sto2}) + k_{gri} \cdot Q_{sto1} \tag{34}$$

$$\frac{d}{dt}(Q_{gut}) = (-k_{abs} \cdot Q_{gut}) + k_{empt} \cdot Q_{sto2} \tag{35}$$

$$\frac{d}{dt}(I_1) = -k_i \cdot (I_1 - I) \tag{36}$$

$$\frac{d}{dt}(I_d) = -k_i \cdot (I_d - I_1) \tag{37}$$

$$\frac{d}{dt}(INS_f) = (-p_{2U} \cdot INS_f) + p_{2U} \cdot (I - I_b) \tag{38}$$

$$\frac{d}{dt}(I_{po}) = (-gamma \cdot I_{po}) + S_{po} \tag{39}$$

$$\frac{d}{dt}(Y) = -alpha \cdot (Y - beta \cdot (G - G_b)) \tag{40}$$

$$\frac{d}{dt}(INS) = V_1 - V_2 \tag{41}$$

$$\frac{d}{dt}(Glu_{in}) = p1 \cdot (V_{in} - V_{out}) - V_{G6P} \tag{42}$$

$$\frac{d}{dt}(G6P) = V_{G6P} - V_{met} \tag{43}$$

All variables of final model:

$$aa = \frac{5/2}{(1-b)/D} \tag{44}$$

$$cc = \frac{5/2}{d/D} \tag{45}$$

$$EGP = k_{p1} - k_{p2} \cdot G_p - k_{p3} \cdot I_d - k_{p4} \cdot I_{po} \tag{46}$$

$$V_{lmax} = V_l + V_{lX} \cdot INS \tag{47}$$

$$V_{mmax} = V_m + V_{mX} \cdot INS \tag{48}$$

$$E = 0 \tag{49}$$

$$S = gamma \cdot I_{po} \tag{50}$$

$$I = \frac{I_p}{V_I} \tag{51}$$

$$G = \frac{G_p}{V_G} \tag{52}$$

$$HE = (-m_5 \cdot S) + m_6 \tag{53}$$

$$m_3 = HE \cdot \frac{m_1}{(1-HE)} \tag{54}$$

$$Q_{sto} = Q_{sto1} + Q_{sto2} \tag{55}$$

$$Ra = f \cdot k_{abs} \cdot \frac{Q_{gut}}{BW} \tag{56}$$

$$k_{empt} = k_{min} + \frac{(k_{max} - k_{min})}{2} \cdot (tanh(aa \cdot (Q_{sto} - b \cdot D)) - tanh(cc \cdot (Q_{sto} - d \cdot D)) + 2) \tag{57}$$

$$bf = (be + kbf \cdot (INS_f + INS_{\text{offset}})) \cdot bradykinin \tag{58}$$

$$bfe_f = (bf - bf_b) \cdot (INS_f - INS_b) \cdot p_{bf} \tag{59}$$

$$S_{po} = Y + K \cdot \frac{(EGP + Ra - E - U_{ii} - k_1 \cdot G_p + k_2 \cdot G_t)}{V_G} + S_b \tag{60}$$

$$INS_{fe} = nC \cdot (k8 \cdot \frac{GLUT4m}{pf} + \frac{GLUT1}{pf} + bfe_f) \tag{61}$$

$$V_{in} = p4 \cdot G_t \cdot INS_{fe} \tag{62}$$

$$V_{out} = p3 \cdot Glu_{in} \tag{63}$$

$$U_{idm} = V_{mmax} \cdot \frac{G_t}{(K_m + G_t)} \tag{64}$$

$$U_{idl} = V_{lmax} \cdot \frac{G_t}{(K_l + G_t)} \tag{65}$$

$$U_{idf} = p_5 \cdot (V_{in} - V_{out}) \tag{66}$$

$$U_{id} = U_{idf} + U_{idm} + U_{idl} \tag{67}$$

$$U = U_{ii} + U_{id} + U_{idl} \tag{68}$$

$$V_2 = k_2 \cdot INS \tag{69}$$

$$V_1 = k_1 \cdot (I - I_b) \tag{70}$$

$$v1a = IR \cdot k1a \cdot (INS_f + 5) \cdot 1e-3 \tag{71}$$
$$v1basal = k1basal \cdot IR \tag{72}$$
$$v1c = IRins \cdot k1c \tag{73}$$
$$v1d = IRp \cdot k1d \tag{74}$$
$$v1e = IRip \cdot k1f \cdot Xp \tag{75}$$
$$v1g = IRp \cdot k1g \tag{76}$$
$$v1r = IRi \cdot k1r \tag{77}$$
$$v2a = IRS1 \cdot k2a \cdot IRip \tag{78}$$
$$v2b = IRS1p \cdot k2b \tag{79}$$
$$v2c = IRS1p \cdot k2c \cdot mTORC1a \cdot diabetes \tag{80}$$
$$v2d = IRS1p307 \cdot k2d \tag{81}$$
$$v2f = IRS1p307 \cdot k2f \tag{82}$$
$$v2basal = IRS1 \cdot k2basal \tag{83}$$
$$v2g = IRS1307 \cdot k2g \tag{84}$$
$$v3a = X \cdot k3a \cdot IRS1p \tag{85}$$
$$v3b = Xp \cdot k3b \tag{86}$$
$$v5a = mTORC1 \cdot (k5a1 \cdot PKB308p473p + k5a2 \cdot PKB308p) \tag{87}$$
$$v5b = mTORC1a \cdot k5b \tag{88}$$
$$v5c = mTORC2 \cdot k5c \cdot IRip \tag{89}$$
$$v5d = k5d \cdot mTORC2a \tag{90}$$
$$v4a = k4a \cdot PKB \cdot IRS1p \tag{91}$$
$$v4b = k4b \cdot PKB308p \tag{92}$$
$$v4c = k4c \cdot PKB308p \cdot mTORC2a \tag{93}$$
$$v4e = k4e \cdot PKB473p \cdot IRS1p307 \tag{94}$$
$$v4f = k4f \cdot PKB308p473p \tag{95}$$
$$v4h = k4h \cdot PKB473p \tag{96}$$
$$v6f1 = AS160 \cdot (k6f1 \cdot PKB308p473p + k6f2 \cdot \frac{PKB473p^n6}{(km6^n6 + PKB473p^n6))} \tag{97}$$
$$v6b1 = AS160p \cdot k6b \tag{98}$$
$$v7f = GLUT4 \cdot k7f \cdot AS160p \tag{99}$$
$$v7b = GLUT4m \cdot k7b \tag{100}$$
$$v9f1 = S6K \cdot k9f1 \cdot \frac{mTORC1a^n9}{km9^n9 + mTORC1a^n9} \tag{101}$$
$$v9b1 = S6Kp \cdot k9b1 \tag{102}$$
$$v9f2 = S6 \cdot k9f2 \cdot S6Kp \tag{103}$$
$$v9b2 = S6p \cdot k9b2 \tag{104}$$
$$V_{G6P} = V_{G6P_{max}} \cdot \frac{Glu_{in}}{(k_{gluin} + Glu_{in})} \cdot \frac{1}{(k_{G6P} + G6P)} \tag{105}$$
$$V_{met} = p3 \cdot G6P \tag{106}$$

Annotation	Value	Identification
diabetes	1	(Brännmark et al. 2013)
k1a	0.633141	(Brännmark et al. 2013)
k1basal	0.0331338	(Brännmark et al. 2013)
k1c	0.876805	(Brännmark et al. 2013)
k1d	31.012	(Brännmark et al. 2013)
k1f	1839.58	(Brännmark et al. 2013)
k1g	1944.11	(Brännmark et al. 2013)
k1r	0.547061	(Brännmark et al. 2013)
k2a	3.22728	(Brännmark et al. 2013)
k2c	5758.78	(Brännmark et al. 2013)
k2basal	0.0422768	(Brännmark et al. 2013)
k2b	3424.35	(Brännmark et al. 2013)
k2d	280.753	(Brännmark et al. 2013)
k2f	2.9131	(Brännmark et al. 2013)
k2g	0.267089	(Brännmark et al. 2013)
k3a	0.00137731	(Brännmark et al. 2013)
k3b	0.0987558	(Brännmark et al. 2013)
k4a	5790.17	(Brännmark et al. 2013)
k4b	34.7965	(Brännmark et al. 2013)
k4c	4.45581	(Brännmark et al. 2013)
k4e	42.8395	(Brännmark et al. 2013)
k4f	143.597	(Brännmark et al. 2013)
k4h	0.536145	(Brännmark et al. 2013)
k5a1	1.8423	(Brännmark et al. 2013)
k5a2	0.055064	(Brännmark et al. 2013)
k5b	24.826	(Brännmark et al. 2013)
k5d	1.06013	(Brännmark et al. 2013)
km5	2.64988	(Brännmark et al. 2013)
k5c	0.0857515	(Brännmark et al. 2013)
k6f1	2.65168	(Brännmark et al. 2013)
k6f2	36.9348	(Brännmark et al. 2013)
km6	30.5424	(Brännmark et al. 2013)
n6	2.13707	(Brännmark et al. 2013)
k6b	65.1841	(Brännmark et al. 2013)
k7f	50.9829	(Brännmark et al. 2013)
k7b	2285.97	(Brännmark et al. 2013)
k8	724.242	(Brännmark et al. 2013)
glut1	7042.19	(Brännmark et al. 2013)
k9f1	0.12981	(Brännmark et al. 2013)
k9b1	0.0444092	(Brännmark et al. 2013)
k9f2	3.3289	(Brännmark et al. 2013)
k9b2	30.9967	(Brännmark et al. 2013)
km9	5872.68	(Brännmark et al. 2013)
n9	0.985466	(Brännmark et al. 2013)
kbf	0.01	(Brännmark et al. 2013)
nC	2.1e-06	(Brännmark et al. 2013)
INS_{offset}	7	(Brännmark et al. 2013)

Table S1: Parameters of adipocyte module.

**Annotation**	**Value**	**Identification**
V_G	1.88	(Dalla Man et al. 2007)
k_1	0.065	(Dalla Man et al. 2007)
k_2	0.079	(Dalla Man et al. 2007)
G_b	95	(Dalla Man et al. 2007)
V_I	0.05	(Dalla Man et al. 2007)
m_1	0.19	(Dalla Man et al. 2007)
m_2	0.484	(Dalla Man et al. 2007)
m_4	0.194	(Dalla Man et al. 2007)
m_5	0.0304	(Dalla Man et al. 2007)
m_6	0.6471	(Dalla Man et al. 2007)
HE_b	0.6	(Dalla Man et al. 2007)
I_b	25	(Dalla Man et al. 2007)
S_b	1.8	(Dalla Man et al. 2007)
k_{max}	0.0558	(Dalla Man et al. 2007)
k_{min}	0.008	(Dalla Man et al. 2007)
k_{abs}	0.057	(Dalla Man et al. 2007)
k_{gri}	0.0558	(Dalla Man et al. 2007)
f	0.9	(Dalla Man et al. 2007)
b	0.82	(Dalla Man et al. 2007)
d	0.01	(Dalla Man et al. 2007)
BW	78	(Dalla Man et al. 2007)
k_{p1}	2.7	(Dalla Man et al. 2007)
k_{p2}	0.0021	(Dalla Man et al. 2007)
k_{p3}	0.009	(Dalla Man et al. 2007)
k_{p4}	0.618	(Dalla Man et al. 2007)
k_i	0.0079	(Dalla Man et al. 2007)
U_{ii}	1.47	(Dalla Man et al. 2007)
p_{2U}	0.0331	(Dalla Man et al. 2007)
K	2.3	(Dalla Man et al. 2007)
$alpha$	0.05	(Dalla Man et al. 2007)
$beta$	0.1	(Dalla Man et al. 2007)
$gamma$	0.5	(Dalla Man et al. 2007)
k_{e10}	0.0005	(Dalla Man et al. 2007)
k_{e2}	339	(Dalla Man et al. 2007)
D	78000	(Dalla Man et al. 2007)

Table S2: Parameters of whole-body module in M0.

Annotation	Value	Identification
V_G	1.88	(Dalla Man et al. 2007)
k_1	0.065	(Dalla Man et al. 2007)
k_2	0.079	(Dalla Man et al. 2007)
G_b	95	(Dalla Man et al. 2007)
V_I	0.05	(Dalla Man et al. 2007)
m_1	0.19	(Dalla Man et al. 2007)
m_2	0.484	(Dalla Man et al. 2007)
m_4	0.194	(Dalla Man et al. 2007)
m_5	0.0304	(Dalla Man et al. 2007)
m_6	0.6471	(Dalla Man et al. 2007)
HE_b	0.6	(Dalla Man et al. 2007)
I_b	25	(Dalla Man et al. 2007)
S_b	1.8	(Dalla Man et al. 2007)
k_{max}	0.0558	(Dalla Man et al. 2007)
k_{min}	0.008	(Dalla Man et al. 2007)
k_{abs}	0.057	(Dalla Man et al. 2007)
k_{gri}	0.0558	(Dalla Man et al. 2007)
f	0.9	(Dalla Man et al. 2007)
b	0.82	(Dalla Man et al. 2007)
d	0.01	(Dalla Man et al. 2007)
BW	78	(Dalla Man et al. 2007)
k_{p1}	2.7	(Dalla Man et al. 2007)
k_{p2}	0.0021	(Dalla Man et al. 2007)
k_{p3}	0.009	(Dalla Man et al. 2007)
k_{p4}	0.618	(Dalla Man et al. 2007)
k_i	0.0079	(Dalla Man et al. 2007)
V_m	2.5	(Nyman et al. 2011)
V_{mx}	0.047	(Nyman et al. 2011)
K_m	225.59	(Nyman et al. 2011)
p_{2U}	0.0331	(Dalla Man et al. 2007)
K	2.3	(Dalla Man et al. 2007)
$alpha$	0.05	(Dalla Man et al. 2007)
$beta$	0.1	(Dalla Man et al. 2007)
$gamma$	0.5	(Dalla Man et al. 2007)
k_{e10}	0.0005	(Dalla Man et al. 2007)
k_{e2}	339	(Dalla Man et al. 2007)
D	78000	(Dalla Man et al. 2007)
$part_f$	0.5	estimated, by hand
$part_m$	0.44	estimated, by hand
$part_l$	1.67	estimated, by hand
U_{ii}	0.8447	estimated, by hand

Table S3: Parameters of whole-body module in M1 and M2a.

annotation	description	value	identification	
U_{ii}	insulin independent glucose uptake	0.831	estimated	
$k2$	rate constant	0.0429	estimated	
$k1$	rate constant	0.0476	estimated	
V_m	basal rate of glucose utilization in muscle tissue	0.881	estimated	
V_{mx}	maximum rate of glucose entering muscle tissue	0.0409	estimated	
K_m	Michaelis-Menten parameter muscle tissue glucose uptake	476	estimated	
V_l	basal rate of glucose utilization in liver tissue	2.00	estimated	
V_{lx}	maximum rate of glucose entering liver tissue	0.0439	estimated	
K_l	Michaelis-Menten parameter liver tissue glucose uptake	355	estimated	
$p1$	rate constant	0.179	estimated	
$p2$	rate constant	4.48	estimated	
$p3$	transport parameter	0.161	estimated	
$p4$	transport parameter	2.63	estimated	
k_{gluin}	Michaelis-Menten parameter for glucose phosphorylation	2.14	estimated	
k_{G6P}	Michaelis-Menten parameter for glucose phosphorylation	11495	estimated	
V_{G6Pmax}	maximum rate of intracellular phosphorylation of glucose	410	estimated	
bf_b	basal blood flow	0 or 3	estimated, by hand	
$bradykinin$	insulin independent glucose uptake	1 or 2.2	estimated, by hand	
be	direct effect of Bradykinin on blood flow	3	estimated, by hand	
p_{bf}	scaling parameter	5	estimated, by hand	
INS_b	basal insulin level in adipose tissue	0.8549	estimated, by hand	

Table S4: Parameters used in module M2b, M3, and M4.

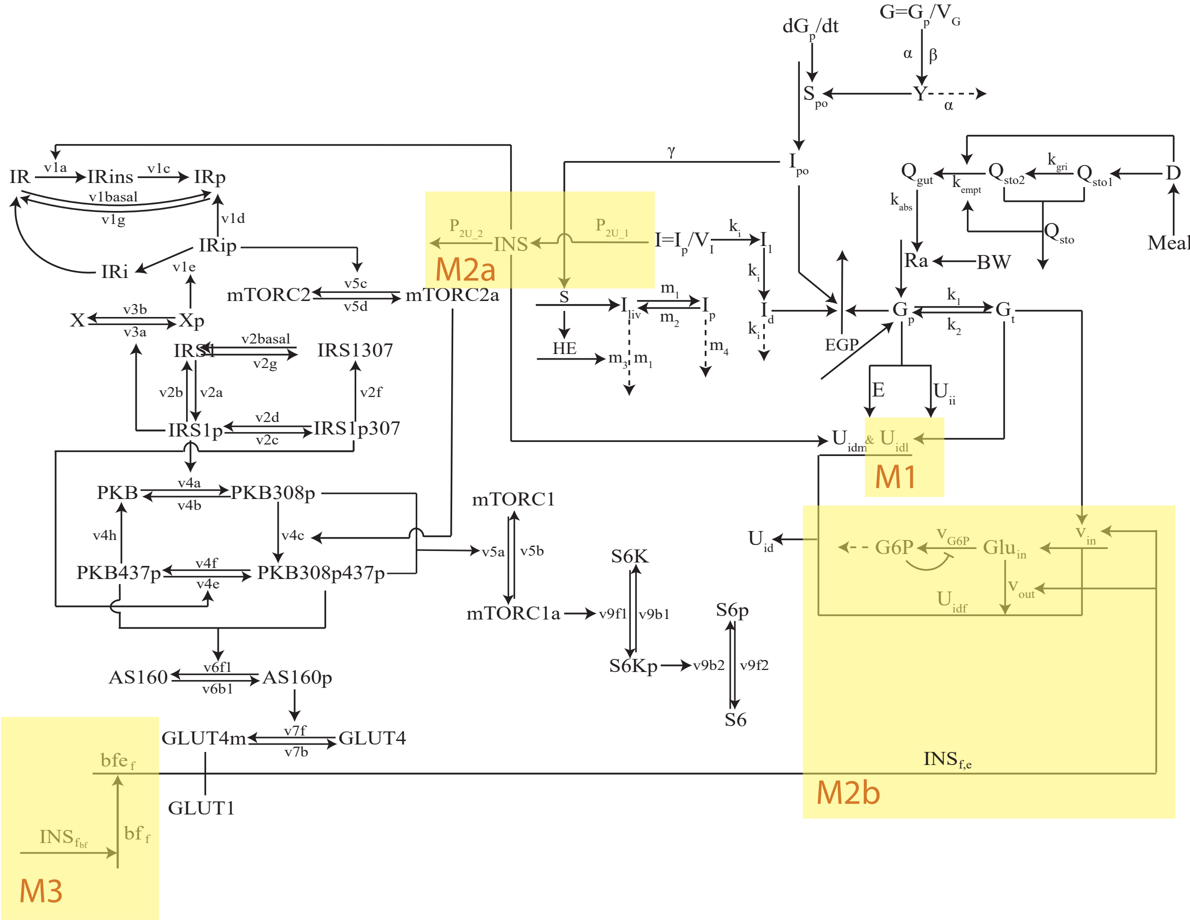

Figure S1: Interaction graph of model M4.

Annotation	value
$IR(0)$	99.10
$IRp(0)$	0.002001
$IRins(0)$	0.7625
$IRip(0)$	0.0199
$IRi(0)$	0.1134
$IRS1(0)$	82.35
$IRS1p(0)$	0.001215
$IRS1p307(0)$	0.3872
$IRS1307(0)$	17.26
$X(0)$	99.998
$Xp(0)$	0.001695
$PKB(0)$	66.92
$PKB308p(0)$	13.26
$PKB473p(0)$	17.71
$PKB308p473p(0)$	2.112
$mTORC1(0)$	84.31
$mTORC1a(0)$	15.69
$mTORC2(0)$	99.84
$mTORC2a(0)$	0.1607
$AS160(0)$	81.92
$AS160p(0)$	18.08
$GLUT4m(0)$	28.73
$GLUT4(0)$	71.27
$S6K(0)$	99.16
$S6Kp(0)$	0.8417
$S6(0)$	91.71
$S6p(0)$	8.290
$Glu_{in}(0)$	3.061
$G6P(0)$	0.004693
$G_p(0)$	178
$G_t(0)$	130
$I_l(0)$	4.5
$I_p(0)$	1.25
$Q_{sto1}(0)$	78000
$Q_{sto2}(0)$	0
$Q_{gut}(0)$	0
$I_1(0)$	25
$I_d(0)$	25
$INS_f(0)$	0
$INS(0)$	0
$I_{po}(0)$	3.6
$Y(0)$	0
$E(0)$	0

Table S5: Initial values.

Full-length name	Abbrevation
Type 1 diabetes	T1D
Type 2 diabetes	T2D
Area under the curve	AUC
insulin receptor	IR
Tyrosine phosphorylation	YP
insulin receptor substrate-1	IRS1
Akt-substrate 160	AS160
S6 kinase beta-1	S6K1
Extracellular signal-regulated kinases	Erk1
ETS Like-1 protein Elk-1	Elk1
Forkhead box protein O1	FOXO1
Endogenous glucose production	EGP
Arteriovenous	AV
Ordinary differential equations	ODEs

Table S6: Abbrevations.
